# Computational evaluation of halogen-bonded cocrystals enables prediction of their mechanochemical interconversion reactions†

**DOI:** 10.1039/d2sc06770f

**Published:** 2023-02-08

**Authors:** Lavanya Kumar, Katarina Leko, Vinko Nemec, Damian Trzybiński, Nikola Bregović, Dominik Cinčić, Mihails Arhangelskis

**Affiliations:** a Faculty of Chemistry, University of Warsaw 1 Pasteura St. 02-093 Warsaw Poland m.arhangelskis@uw.edu.pl; b Faculty of Science, Department of Chemistry, University of Zagreb Horvatovac 102a HR-10000 Zagreb Croatia; c Biological and Chemical Research Centre, University of Warsaw Żwirki i Wigury 101 02-089 Warsaw Poland

## Abstract

Periodic density-functional theory (DFT) calculations were used to predict the thermodynamic stability and the likelihood of interconversion between a series of halogen-bonded cocrystals. The outcomes of mechanochemical transformations were in excellent agreement with the theoretical predictions, demonstrating the power of periodic DFT as a method for designing solid-state mechanochemical reactions prior to experimental work. Furthermore, the calculated DFT energies were compared with experimental dissolution calorimetry measurements, marking the first such benchmark for the accuracy of periodic DFT calculations in modelling transformations of halogen-bonded molecular crystals.

## Abstract 2

Periodic density-functional theory (DFT) calculations and dissolution calorimetry measurements were used to predict the thermodynamic stability and the likelihood of interconversion between a series of halogen-bonded cocrystals.

## Graphical abstract

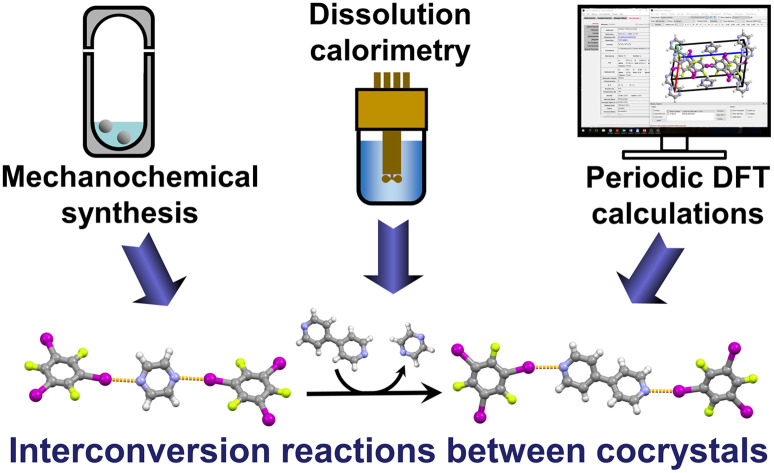

## 1. Introduction

Cocrystallization, as a route to construct multicomponent materials containing neutral molecules has become a prominent method for achieving property-driven materials design, with applications in pharmaceutical crystal form screening,^1–4^ luminescent materials,^5–7^ molecular semiconductors,^8^ thermochromic devices^9,10^ and energetic materials.^11,12^ A successful cocrystallization experiment relies on complementarity of the constituent molecules, manifested through the formation of strong and robust supramolecular interactions. Design of cocrystals therefore strongly relies on the possibility of forming robust supramolecular synthon^13^ interactions between constituent molecules, with computational methods becoming increasingly widely used in virtual screening studies preceding experimental screening.^14–16^ While hydrogen bonding (HB) has been the most widely used interaction to construct cocrystals from molecules containing HB donor and acceptor sites,^17–19^ its use is largely limited to elements from the second and third periods of the periodic table. Conversely, a family of interactions,^20^ based on a positive electrostatic potential region (σ-hole) located on the donor atom, which includes tetrel,^21,22^ pnictogen,^23^ chalcogen^24–26^ and, finally, halogen bonds,^27–30^ presents a route towards supramolecular functionalization of heavy elements, all the way to the sixth row of the periodic table.^31^ In terms of functional properties, halogen bonding has been used in molecular recognition,^32^ design of phosphorescent materials^33^ and pharmaceutical crystal engineering.^34^

Successful design and synthesis of halogen-bonded materials^35^ relies on an accurate understanding of their thermodynamic stability with respect to individual starting materials or competing structures (polymorphs,^36^ cocrystals of different stoichiometry,^37^ cocrystal solvates^38^). In our previous work we have demonstrated how periodic density-functional theory (DFT) calculations enable quantitative predictions of cocrystal mechanochemical reactivity through assessment of thermal stability,^39^ stoichiometric interconversions,^40^ topological transformations^41^ and formation of unprecedented halogen bonds with P,^42^ As and Sb acceptor atoms.^43^ In all of these studies we have demonstrated that periodic DFT calculations provide a quantitative evaluation of the thermodynamics of solid-state mechanochemical transformations involving halogen-bonded solids. Crucially, the periodic nature of these calculations allows for consideration of the crystal lattice as a whole, taking into account the energetic effects of all supramolecular interactions present in the crystal lattice, not just the halogen bonds. Thus, the overall thermodynamic driving force for the crystal lattice transformations can be calculated in order to predict the likelihood of the corresponding solid-state mechanochemical processes occurring experimentally.

The accuracy of periodic DFT calculations has been previously verified^44–46^ in predicting the polymorph stability and explaining the sequences of mechanochemical polymorph interconversions of metal–organic frameworks (MOFs) with the aid of dissolution calorimetry measurements. Such experimental benchmarks provide an excellent opportunity to study the limits of theoretical models, verify and improve their accuracy. In this work we will utilize the combination of periodic DFT calculations and calorimetric measurements to predict the outcomes of mechanochemical transformations of halogen-bonded cocrystals, as a way to computationally screen for the transformations that are likely to occur, before performing these reactions experimentally *via* mechanochemistry.^47–49^

## 2. Experimental and computational methods

### 2.1. Periodic DFT calculations

Periodic DFT calculations were performed using the plane-wave DFT code CASTEP 20.^50^ The CIFs of the crystal structures of individual cocrystal components and cocrystal structures, obtained from the Cambridge Structural Database,^51^ were converted to CASTEP input format using cif2 cell.^52^ The crystal structures were then geometry-optimized with respect to unit cell parameters and atom coordinates, subject to crystallographic symmetry constraints. The calculations were performed with PBE functional, combined with either Grimme D3 (ref. 53) or many-body dispersion (MBD*)^54–56^ correction. The plane-wave cutoff was set to 800 eV. Further details of the periodic DFT calculations, together with the calculated energies of the individual crystal structures are given in ESI Section S2.†

### 2.2. Solution crystallization

The single crystals of the pyrazine cocrystal with 1,3,5-trifluoro-2,4,6-triiodobenzene (pyr)_1/2_(tftib), were obtained by slow evaporation of a solution of tftib (60 mg, 0.118 mmol) and pyr (16 mg, 0.236 mmol) in 1 ml of acetonitrile. The resulting single crystals were used for crystal structure determination by single crystal X-ray diffraction (XRD). Details of XRD measurements, structure solution and refinement methods are given in ESI Section S3.4.†

### 2.3. Mechanochemical synthesis and interconversion of cocrystals

All mechanochemical ball milling reactions were performed using Retsch MM-400 shaker mill. In a typical experiment the starting materials were mixed in the stoichiometric ratio, according to the reaction equation, with the total mass of the reactants of 200 mg. The reactants were placed inside a 10 ml milling jar, with the addition of 40 μl (*η* = 0.20 μl mg^−1^)^57^ of one of the liquid additives (ethanol, acetonitrile or hexane), together with two stainless steel 7 mm balls. The reaction mixture was milled at 30 Hz frequency for 30 minutes. In selected cases, duration of the milling experiments was extended to 1 hour, see ESI† for details.

The products of mechanochemical reactions were analyzed by powder X-ray diffraction (PXRD), and the quantitative compositions were determined using Rietveld refinement^58^ quantitative phase analysis (see ESI† Section S1.1 and S3.1 for details).

### 2.4. Competitive slurry experiments

Competitive slurry experiments were performed by stirring a mixture of reaction materials (150 mg) suspended in 40 μl of acetonitrile (*η* = 0.27 μl mg^−1^), sealed in a glass vial. The compositions of the product mixtures after slurrying were analyzed by PXRD and Rietveld refinement (see ESI Section S1.2† for details).

### 2.5. Dissolution calorimetry

Dissolution calorimetry measurements were performed on a TA Instruments TAM IV calorimeter. Pre-weighed solid samples were exposed to 15 ml acetonitrile, pre-equilibrated at 25 °C temperature. Enthalpy of dissolution for each material was measured in duplicate or triplicate. For details of the measurement procedure see ESI Section S3.5.†

### 2.6. Thermal analysis

Differential scanning calorimetry (DSC) and thermogravimetric analysis (TGA) measurements were performed on a Mettler-Toledo TGA/DSC STAR^e^ instrument. Pre-weighed samples (approx. 10 mg) were placed in alumina crucibles, and heated up to 300 °C temperature at a rate of 10 °C min^−1^, under nitrogen flow.

## 3. Results and discussion

**Fig. 1:**
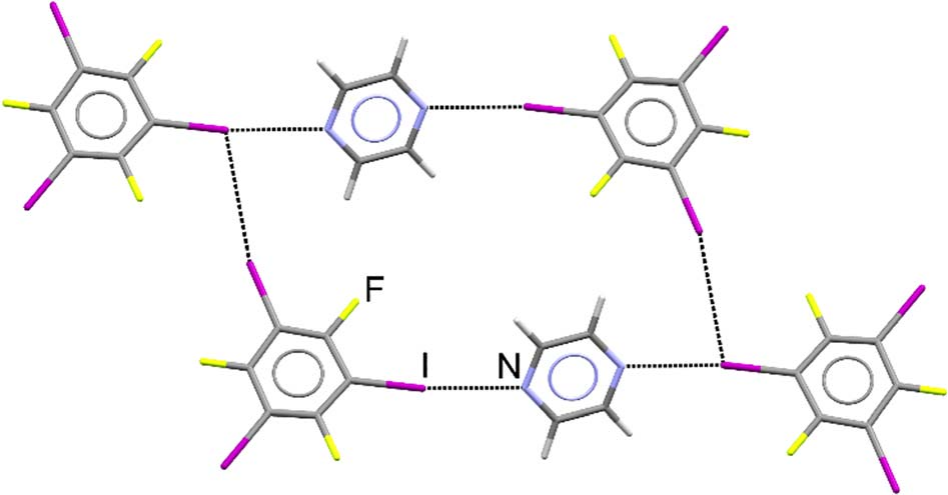
Structure of the cocrystal of pyrazine with 1,3,5-trifluoro-2,4,6-triiodobenzene, further labelled as (pyr)_1/2_(tftib). Intermolecular N⋯I and I⋯I interactions are shown.

The periodic DFT calculations and dissolution calorimetry measurements,^59,60^ which are the core of this study, were motivated by the initial preparation of a new cocrystal of pyrazine (pyr) with 1,3,5-trifluoro-2,4,6-triiodobenzene (tftib) (Fig. 1), which was synthesized mechanochemically, using liquid-assisted grinding (LAG)^57^ with ethanol. Upon recrystallization from solution, single crystals suitable for diffraction analysis were obtained, revealing that the cocrystal has (pyr)_1/2_(tftib) stoichiometry. Both nitrogen atoms of pyr are utilized in N⋯I halogen bonding interactions to two distinct tftib donor molecules. Conversely, only one out of three iodine atoms of tftib is used to form N⋯I halogen bonds, with the other two iodines involved in I⋯I and I⋯F interactions.^37^

**Fig. 2:**
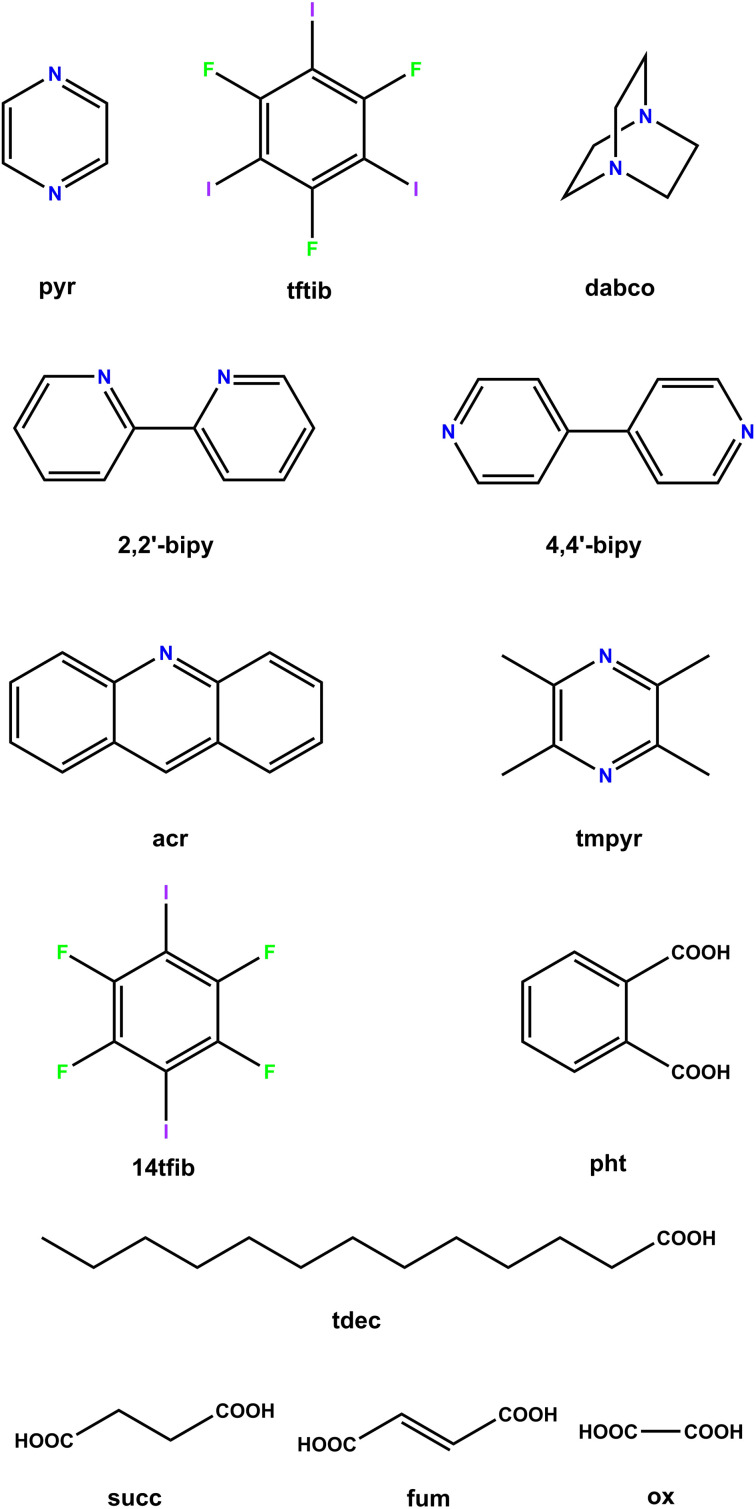
Molecular diagrams of the cocrystal components used in this work.

Since certain cocrystals of pyr have been found to undergo dissociation under relatively mild conditions,^61^ and pyr itself is a volatile material,^62^ it was decided to investigate the stability and reactivity of the (pyr)_1/2_(tftib) cocrystal more closely, in comparison to other previously reported cocrystals where tftib acts as a halogen-bond donor in combination with different acceptor molecules (Fig. 2).

Periodic DFT calculations using the PBE^63^ functional combined with either Grimme D3 (ref. 53) or many-body dispersion (MBD*)^54–56^ semiempirical correction schemes were performed for the cocrystal structures, as well as for the corresponding individual component structures, allowing us to derive the energies for cocrystal interconversion reactions, where pyr is displaced from the (pyr)_1/2_(tftib) cocrystal by a competing coformer. The reaction equations, with their calculated energies are summarized in Table 1 (reactions 1–5). Importantly, the energies calculated with PBE + D3 and PBE + MBD* methods were in good agreement, increasing our confidence in the accuracy of these results. Most crucially, for all but one of the halogen bond acceptor exchange reactions (1–5), except for the reaction with acr (reaction 4) the interconversion energies were found to be negative, suggesting that such reactions are likely to occur experimentally.^40^

**Table 1:** Energies for the cocrystal interconversion reactions calculated from periodic DFT and calorimetry data, as well as experimental outcomes of the corresponding mechanochemical transformations. The average differences between the energies calculated with two DFT methods are: MAE 2.1 kJ mol^−1^; RMSD 2.2 kJ mol^−1^

Reaction Nr	Reaction type	Reaction equation	Δ*E*/kJ mol^−1^			Experimental LAG reactions (forward/reverse)		
			PBE + D3	PBE + MBD*	Experimental calorimetry	Ethanol	Aceto-nitrile	Hexane
1	Acceptor exchange	(pyr)_1/2_(tftib) + 2,2′-bipy → (2,2′-bipy)(tftib) + 1/2 pyr	−3.4	−4.7	−5.7(8)	Forward	Forward	Forward^a^
2		(pyr)_1/2_(tftib) + 4,4′-bipy → (4,4′-bipy) (tftib) + 1/2 pyr	−18.4	−20.1	−13.2(6)	Forward	Forward	Forward
3		(pyr)_1/2_(tftib) + dabco → (dabco)(tftib) + 1/2 pyr	−22.3	−18.3	−10.0(8)	Forward	Forward	Forward^a^
4		(pyr)_1/2_(tftib) + acr → (acr) (tftib) + 1/2 pyr	−1.0	0.2	0.4(5)	Forward	Forward	Forward^b^
5		(pyr)_1/2_(tftib) + tmpyr → (tmpyr)(tftib) + 1/2 pyr	−8.1	−6.0	−7.1(7)	Forward	Forward	Forward
6	Donor exchange	(pyr)_1/2_(tftib) + ½ 14tfib → 1/2 (pyr)(14tfib) + tftib	0.3	−1.4	0.6(7)	Reverse	Reverse	Reverse
7		(pyr)_1/2_(tftib) + 1/2 fum → 1/2 (pyr)(fum) + tftib	6.9	5.1	4.2(5)	Reverse	Reverse	Reverse
8		(pyr)_1/2_(tftib) + 1/2 pht → 1/2 (pyr)(pht) + tftib	12.3	9.5	6.8(7)	Reverse	Reverse	Reverse
9		(pyr)_1/2_(tftib) + tdec → 1/2 (pyr) (tdec)_2_ + tftib	6.1	9.2	4.7(8)	Reverse	Reverse	Reverse^c^
10		(pyr)_1/2_(tftib) + 1/2 succ → 1/2 (pyr)(succ) + tftib	7.3	5.5	1.3(1)	Reverse	Reverse	Reverse^c^
11		(pyr)_1/2_(tftib) + 1/2 ox → 1/2 (pyr)(ox) + tftib	−3.6	−5.1	−1.8(5)	Forward	Forward	Forward

a Reaction proceeds with a solvated intermediate.

b LAG reactions result in a mixture of (pyr)_1/2_(tftib) and (acr)(tftib), upon storage the proportion of (acr)(tftib) in the reaction mixture increases (ESI Fig. S6 and S7).

c LAG reactions result in a mixture of (pyr)_1/2_(tftib) and product cocrystal for the corresponding reaction, but full conversion to the product cocrystal is observed upon storing the samples at room temperature up to 3 months.

**Fig. 3:**
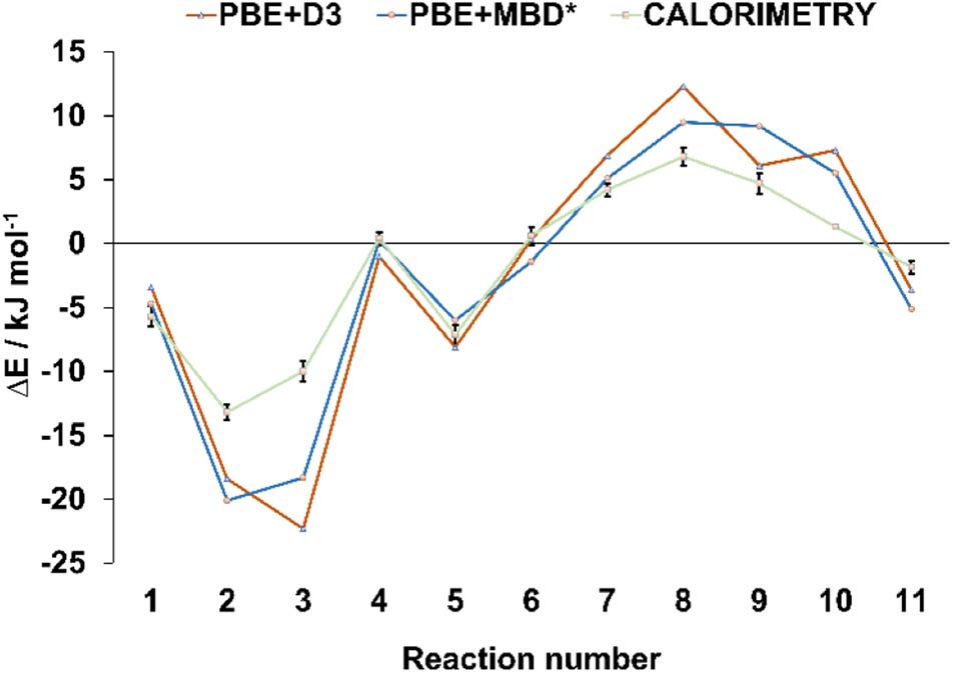
Comparison of the theoretical and experimental energies for the interconversion reactions from Table 1. The reaction energies are represented by triangular and square markers, with error bars shown for the energies measured experimentally, *via* dissolution calorimetry. The lines connecting the markers were added only for better visibility, they do not represent any specific trends.

In order to verify the validity of the cocrystal reactivity predictions made with periodic DFT, we performed dissolution calorimetry measurements for the cocrystal materials and their individual components. The pre-weighed materials were dissolved in acetonitrile in the calorimetric cell, and the heat effect of dissolution was measured. The average dissolution enthalpies from duplicate or triplicate measurements were used in subsequent calculations. The measured dissolution enthalpies were then used to construct thermodynamic cycles, which provided the experimental enthalpies of cocrystal interconversion reactions, for direct comparison with periodic DFT predictions. The experimental uncertainties for the measured reaction energies were consistently within +/− 1 kJ mol^−1^, which is less than the typical difference between the reaction energies calculated with the two periodic DFT methods (average absolute difference 2.1 kJ mol^−1^). Such low experimental uncertainty therefore allows for quantitative validation of the accuracy of the periodic DFT calculations. The comparison revealed excellent agreement between the calculated and measured enthalpies of cocrystal interconversion reactions, as can be seen in Table 1 and Fig. 3. In particular, DFT and calorimetry consistently show which reactions are strongly exothermic and thus thermodynamically highly favored (reactions 2, 3), while also reliably predicting the borderline cases with reaction enthalpies close to zero (reaction 4, involving acr). Borderline cases, where reaction energy is close to zero pose a challenge for periodic DFT, since the sign of the reaction energy is being used as a predictor for the experimental occurrence of the reaction. In such cases the reaction entropy can play the decisive role in determining the relative thermodynamic stability of the crystal phases and, consequently the direction of the reaction.^64^ In the majority of cases, however, the calculated reaction energies exceed +/−2 kJ mol^−1^, such that periodic DFT calculations serve as an accurate predictor for the thermodynamic effects of solid-state transformations of halogen-bonded materials. This, in turn, is crucial for being able to use periodic DFT calculations as a way to predict the likelihood of these transformations occurring experimentally. To the best of our knowledge, this is the first example of validating the accuracy of periodic DFT calculations by experimental dissolution calorimetry measurements performed for halogen-bonded molecular crystals.

Having analyzed the thermodynamics of pyrazine displacement reactions by competing halogen bond acceptors, through a combination of periodic DFT and dissolution calorimetry, we proceeded with performing the proposed reactions experimentally using mechanochemistry. In these reactions (pyr)_1/2_(tftib) cocrystal samples were mixed with each of the coformers in a stoichiometric ratio determined by the corresponding reaction equation, and ground using a vibrational ball-mill, under the conditions of liquid-assisted grinding with ethanol additive (see the Experimental section and ESI† for details). Powder X-ray diffraction (PXRD) and Rietveld refinement were used to quantify the composition of reaction products, and verify the formation of cocrystals according to the proposed reaction equations.

Gratifyingly, for all acceptor exchange reactions with predicted negative energies, transformations were observed experimentally. Moreover, when reverse reactions were attempted for all these systems, *i.e.* reactions of cocrystals of alternative coformers with pyr (Δ*E* for those reactions is necessarily positive), none of these reactions had occurred, confirming that the outcome of the reactions was thermodynamically controlled.

Having observed a complete agreement between the experimental and predicted outcomes of cocrystal interconversion reactions, involving displacement of pyr with alternative halogen bond acceptors, we decided to look at the reactions involving displacement of tftib from the (pyr)_1/2_(tftib) cocrystal by alternative donor molecules. First, we have considered a competition between tftib and a closely-related donor 14tfib (Fig. 2). The enthalpy of interconversion (reaction 6 in Table 1) was predicted to be close to zero by both DFT methods and calorimetry, which could be rationalized by the similarity of halogen-bonded interactions found in both cocrystals. Experimentally, the forward reaction was not observed under LAG conditions, while the reverse (*i.e.* (pyr)(14tfib) reacting with tftib to form (pyr)_1/2_(tftib)) occurred in quantitative yield. The outcome of this reaction was difficult to predict by periodic DFT, given a near zero calculated interconversion energy, with the sign dependent on the choice of the method (+0.3 kJ mol^−1^ and −1.4 kJ mol^−1^ for PBE + D3 and PBE + MBD*, respectively). Most importantly, experimental calorimetric measurements also revealed near-zero enthalpy of interconversion for this system, highlighting the agreement between calculated and experimental reaction energies for the transformations between essentially isoenthalpic halogen-bonded cocrystals. Similarly to the previously discussed reaction 4 with a close to zero energy of interconversion, the entropy contribution to the lattice free energy^64^ of reaction 6 may affect the direction it takes.

Having explored the interconversions between halogen-bonded cocrystals with alternative acceptor and donor molecules, we decided to explore competition between halogen and hydrogen bonding interactions,^48,65–69^ and establish whether hydrogen bond donor molecules can displace tftib from (pyr)_1/2_(tftib) cocrystal. Since pyr is known for the propensity to form hydrogen-bonded cocrystals with carboxylic acids,^70–73^ we selected a series of acid coformers, namely ox, succ, fum, pht and tdec (see Fig. 2). Among these coformers, only ox was predicted to displace tftib out of (pyr)_1/2_(tftib) cocrystals, based on both periodic DFT calculations and calorimetric measurements. When the LAG reactions were performed, the results were in complete agreement with the thermodynamic predictions based on periodic DFT and calorimetry. This emphasized the power of our approach in predicting interconversions not only between different halogen-bonded cocrystals, but also for transformations involving a change of interaction type from halogen- to hydrogen bonding.

The experimental reactions discussed so far, were performed under LAG conditions, using ethanol as a liquid additive. In all cases, we have identified the nature of the products by PXRD, ruling out the possibility of solvate formation, and verifying the role of ethanol as a lubricant, rather than an actual component within the crystal structures of either starting materials or products of the interconversion reactions. Nonetheless, in order to further support the generality of our observations, regarding the predictability of reaction outcomes by periodic DFT and calorimetry, we have performed a series of validation experiments, where acetonitrile and hexane were used as a LAG additive^74^ instead of ethanol. In addition, for each cocrystal system we performed competitive slurry experiments in acetonitrile, in order to independently establish their thermodynamic preferences (ESI Table S3†).

All LAG reactions with acetonitrile yielded identical products as the reactions with ethanol liquid additive (see Table 1 and ESI Table S3†). Also, the slurry experiments, as the decisive method for the determination of thermodynamically stable phases,^3,75,76^ showed consistency with DFT calculations, calorimetric measurements and LAG reaction outcomes. In case of hexane-mediated LAG, several reactions were found to proceed *via* intermediate phases of unknown crystal structure (reactions 1 and 3 in Table 1) or result in a mixture of product and reactant cocrystals (reactions 4, 9 and 10 in Table 1). Unknown phases in reactions 1 and 3 were found to be solvated intermediates, as evidenced from TGA weight loss, observed at low temperatures (see ESI Fig. S12–S16†). Both of these solvated intermediates, however, converted to the unsolvated cocrystal products upon storage, as confirmed by PXRD (see ESI† Fig. S2–S5). In three cases, namely reactions 4, 9 and 10, hexane LAG reactions initially led to the formation of reactant and product cocrystal mixtures (ESI Fig. S6, S8 and S10†). Upon storage, the samples corresponding to reactions 9 and 10, converted exclusively to the thermodynamically stable products (ESI Fig. S9 and S11†). In the case of hexane LAG reaction 4 (with acr acceptor), (acr)(tftib) was found as a major product, with additional presence of (pyr)_1/2_(tftib) and tftib phases (ESI Fig. S11†). Reaction 4, however, is a special case, given the calculated and measured interconversion energy is closest to zero, of all reactions considered in this study.

The series of experiments with hexane as a LAG additive confirmed that the use of certain LAG additives may slow down the interconversion process, or lead to formation of transient solvated structures.^77^ However, the final reaction products with hexane liquid additive were identical to those found in ethanol- and acetonitrile-based LAG experiments, as well as in competitive slurry experiments, except for one special case of the reaction with acr.

## 4. Conclusions

We have demonstrated that periodic DFT calculations can be used as a powerful method for predicting solid state reactivity of halogen-bonded cocrystals. We have shown that the calculated reaction enthalpies are a robust indicator of reaction spontaneity under experimental conditions. Such predictions are reliable for reaction enthalpies which exceed +/− 2 kJ mol^−1^, covering all but the most borderline cases. Moreover, quantitatively the interconversion energies calculated with periodic DFT were in good agreement with calorimetric data (Fig. 3), demonstrating the ability to describe the energetics of halogen-bonded cocrystals.

Stability of halogen bonds in solution has previously been investigated using isothermal calorimetric titration method (ITC),^78^ however dissolution calorimetry measurements presented herein, for the first time provide a more direct measure of the energetics of halogen-bonded cocrystals in the solid state. Moreover, dissolution calorimetry provided an experimental proof that the applied computational methods were suitable for describing the investigated reactions, yielding reaction enthalpies directly comparable to the values calculated with periodic DFT.

In conclusion, our work highlights the progress we have made with computational modelling of halogen-bonded materials. The excellent accuracy offered by periodic DFT calculations makes it a valuable method for predicting the outcomes of solid-state reactions before entering the laboratory, thus reducing the number of experimental trials, necessary for crystal form screening, and greatly improving our understanding of solid state reactivity of halogen-bonded cocrystals.

## Data availability

Open access dataset containing experimental and computational data, including PXRD patterns; dissolution calorimetry curves; TGA and DSC curves; periodic DFT calculation input and output files, is available at: https://www.doi.org/10.17605/OSF.IO/7VK5F.

## Author contributions

LK performed synthesis and characterization of the new (pyr)_1/2_(tftib) cocrystal as well as all the mechanochemical cocrystal interconversion reactions and slurry experiments, followed by characterization of the reaction products. DT performed single-crystal X-ray diffraction measurements. KL and NB performed calorimetric measurements. VN and DC contributed to the experimental characterization of synthesized materials. MA designed the project and performed the periodic DFT calculations. All authors contributed to the preparation of the manuscript.

## Conflicts of interest

There are no conflicts to declare.

## Supplementary Material

SC-014-D2SC06770F-s001

SC-014-D2SC06770F-s002
