# Supplementary material for: Computational evaluation of halogen-bonded cocrystals enables prediction of their mechanochemical interconversion reactions
Source: Chem Sci. 2023 Feb 8;14(12):3140–6. doi: 10.1039/d2sc06770f (PMC10033997; doi:10.1039/d2sc06770f)
Supplement: SC-014-D2SC06770F-s001 [file SC-014-D2SC06770F-s001.pdf]

Electronic Supplementary Material (ESI) for Chemical Science.
This journal is © The Royal Society of Chemistry 2023

Computational evaluation of halogen-bonded cocrystals enables prediction of their mechanochemical interconversion reactions

Lavanya Kumar,[a] Katarina Leko,[b] Vinko Nemec,[b] Damian Trzybiński,[c] Nikola Bregović,[b] Dominik Cinčić[b] and Mihails Arhangelskis*[a]

[a] Faculty of Chemistry, University of Warsaw, 1 Pasteura Street, 02-093Warsaw, Poland.

[b] Faculty of Science, Department of Chemistry, University of Zagreb, Horvatovac 102a, HR-10000 Zagreb, Croatia.

[c] Biological and Chemical Research Centre, University of Warsaw, Żwirki i Wigury 101, 02-089 Warsaw, Poland.

*Corresponding author email: m.arhangelskis@uw.edu.pl

Table of Contents

S1 Materials and synthetic procedures 3
S1.1 Mechanochemical synthesis 3
S1.1.1 Synthesis of $(pyr)_{1/2}$(tftib) 3
S1.1.2 Interconversion reactions 3
S1.2 Competitive slurry experiments 4
S2 Periodic density-functional theory calculations 5
S3 Instrumental characterization 6
S3.1 Powder X-ray diffraction measurements 6
S3.2 Rietveld refinement 6
S3.3 Thermogravimetric analysis 6
S3.4 Single crystal X-ray diffraction measurements 6
S3.5 Dissolution calorimetry 7
S4 Results of calculations and experiments 8
S4.1 Periodic DFT calculation results 8
S4.2 Results of mechanochemical and slurry experiments 10
S4.3 Rietveld refinements of reaction products 15
S4.3 Thermal measurements 21
S4.4 Crystallographic information 24
S4.5 Dissolution calorimetry measurements 29
S5 References 47

S1 Materials and synthetic procedures

Most of the reagents and solvents were purchased from **Sigma Aldrich**, unless otherwise stated. The **tftib** reagent was purchased from **Alfa Aesar**. Ethanol and Acetonitrile solvents were purchased from **POCH**.

All the reagents were used without further purification. The only reagent which required further processing before being used in mechanochemical reactions was **ox.2 H_2O**, purchased from **Biomus** , and which had to be dehydrated first. In order to isolate anhydrous **ox**, the dihydrate material was heated to 110 ºC for 3 hours in an oven. The formation of anhydrous **ox** was confirmed by PXRD before using the material for any mechanochemical reactions.

S1.1 Mechanochemical synthesis

Mechanochemical reactions reported here were performed on a 200 mg scale, with reagents placed in 10 ml stainless-steel jars, produced by Retsch. Two stainless steel balls of 7 mm diameter were added to the jar. All reactions were performed under liquid-assisted grinding (LAG) conditions, with the addition of 40 µl of either ethanol, acetonitrile or hexane to the solid reactants. The reaction mixtures were milled for 30 minutes (unless otherwise stated in specific cases) at 30 Hz frequency using a Retsch MM400 shaker mill. The identity of the reaction products was verified using powder X-ray diffraction (PXRD) and Rietveld refinement (see S2.1 and S2.2).

S1.1.1 Synthesis of (pyr)$_{1/2}$(tftib)

Crystals of (**pyr**)$_{1/2}$(**tftib**) suitable for single crystal X-ray diffraction (SCXRD) measurement were obtained by slow evaporation of a saturated solution of **tftib** (95.84 mg, 0.188 mmol) and **pyr** (7.53 mg, 0.094 mmol) in 1 ml acetonitrile. Solid state synthesis of (**pyr**)$_{1/2}$(**tftib**) cocrystal was performed by mixing 185.56 mg (0.364 mmol) of **tftib** and 14.58 mg (0.182 mmol) of **pyr** with the addition of 40 µL of ethanol, and milling the resulting mixture for 30 minutes. The resulting product was quantitatively analyzed by PXRD and Rietveld refinement in order to confirm structural match with the single crystal structure obtained from solution (Figure S1).

S1.1.2 Interconversion reactions

Interconversion reactions in either forward or reverse direction were performed by adding donor/acceptor to (**pyr**)$_{1/2}$(**tftib**) or ½**pyr** to (**acceptor**)$_x$(**tftib**) or **tftib** to (**donor**)$_x$(**pyr**) cocrystals according to the reaction equations from Table 1. The reactions were performed under conditions of ball milling according to procedures described in **S.1.1.1**, with ethanol liquid additive.

Forward reaction with **ox** (Equation 11 in Table 1) and reverse reaction with **tdec** (Equation 9 in Table 1) showed incomplete conversion after 30 mins of milling, however increasing the milling time to one hour resulted in the quantitative formation of the products according to the reaction equations.

In addition, for the reactions not expected to occur based on theoretical thermodynamic predictions, two more sets of experiments were performed, with acetonitrile and hexane liquid additives instead of ethanol.

All the reaction products were identified by PXRD and Rietveld refinement (Table S3).

S1.2 Competitive slurry experiments

In order to identify the thermodynamically stable phase for each reaction system at ambient temperature, we performed competitive slurry experiments for all cocrystal systems discussed in this work. For a typical slurry experiment, a mixture of **tftib**, **pyr**, and an additional donor/acceptor component were mixed in a stochiometric ratio corresponding to the reaction equation (**Table 1**), to a total mass of 150 mg with an addition of 40 μL of acetonitrile. The reaction mixtures were then stirred in a sealed glass vial for 2 hours using a 10 mm stirring bar. The resulting material was dried in open air and the phase composition was determined by PXRD (**Table S3**).

S2 Periodic density-functional theory calculations.

Periodic density-functional theory (DFT) calculations were used to calculate energies for cocrystal formation and interconversion reactions. Calculations were performed in the plane-wave DFT code CASTEP 20.[1] Crystal structures of the cocrystals and individual cocrystal components were obtained from Cambridge Structural Database (CSD)[2] in CIF format, and then converted to CASTEP input program using the cif2cell utility.[3] The DFT calculations were performed with PBE[4] functional combined with either Grimme D3[5] or many-body dispersion (MBD*)[6–8] correction scheme, giving two sets, therefore all reaction energies were calculated with two methods, namely PBE+D3 and PBE+MBD*. The plane-wave basis set was truncated at 800 eV cutoff, while CASTEP default ultrasoft pseudopotentials were used for the core regions of electron density. The 1st electronic Brillouin zone was sampled with a $2\pi x0.07$ Å^{-1} Monkhorst-Pack[9] k-point spacing. All crystal structures were geometry-optimized with respect to unit cell parameters and atom coordinates, considering the space group symmetry constraints. Geometry optimization criteria were set with respect to energy change: $2x10^{-5}$ eV atom^{-1}; maximum atomic force: $5x10^{-2}$ eV Å^{-1}; maximum atom displacement: 10^{-3} Å; maximum residual stress: $5x10^{-2}$ GPa.

S3 Instrumental characterization

S3.1 Powder X-ray diffraction measurements

Powder X-ray diffraction (PXRD) measurements were performed on a Bruker D8 Advance diffractometer with Cu*K*α (λ = 1.54184 Å) radiation source, operating at 40 kV and 40 mA, and equipped with a Lynxeye detector. PXRD patterns were collected in Bragg-Brentano geometry, with a 2θ range of 2/3º and 50º and a step size 0.04º.

S3.2 Rietveld refinement

Rietveld refinement[10] was used to quantify the product composition obtained from mechanochemical and slurry experiments, as shown in **Table S3.** The refinements were performed in TOPAS Academic 7.[11] The background was modelled with a 6th Chebyshev polynomial function, and the diffraction peaks were modelled with a pseudo-Voigt function, coupled with simple axial divergence model. The specimen displacement and unit cell parameters were refined, while the atom coordinates were kept fixed throughout the refinement. When necessary, preferred orientation was accounted for using March-Dollase[12] 1- or 2-directional model.

S3.3 Thermogravimetric analysis

The TGA/DSC analysis was performed using the Mettler-Toledo TGA/DSC STAR^e system. Approximately 10 mg of each sample (accurately weighted using Mettler-Toledo XS105 DualRange balance) were placed into standard 70 µl alumina crucible and heated at 10 ºC/min from 30 to 300 °C, under a constant flow of dry N_2 (60 ml/min).

S3.4 Single crystal X-ray diffraction measurements

Good quality single-crystals of **(pyr)$_{1/2}$(tftib)** was selected for the X-ray diffraction measurement at T = 100(2) K. Diffraction data were collected on the Agilent Technologies SuperNova Single Source diffractometer with Mo*K*α radiation (λ = 0.71073 Å) using CrysAlis software.[13] The spherical absorption correction using equivalent radius and absorption coefficient was applied. The structural determination procedure was carried out using the SHELX package.[14] The structures were solved with direct methods and then successive least-square refinement was carried out based on the full-matrix least-squares method on F^2 using the SHELXL program.[14] The H-atoms were positioned geometrically, with C–H equal to 0.93 and constrained to ride on their parent atoms with U_{iso}(H) = 1.2U_{eq}(C). In case of the phenyl ring of the **tftib** molecule RIGU restraint was applied. Additionally, the C2 and C6 atoms were subject to ISOR restraints. The figures for this publication were prepared using Olex2[15] and Mercury[16] programs.

S3.5 Dissolution calorimetry

The dissolution enthalpy of studied materials in acetonitrile at 25°C was determined calorimetrically by means of TAM IV calorimeter (TA Instruments). The solid samples (m = 0.5 – 11 mg) were placed in sample cartridges and the solvent (15 mL of acetonitrile) was placed in calorimeter cell. The cell was mounted in the calorimeter, the stirring was turned on (stirring rate was 60 rpm) and the system was allowed to reach thermal equilibrium (usually overnight). The related baseline criteria were: heat flow (baseline slope within 30 min) < 500 nW/h, heat flow standard deviation < 100 nW. After baseline equilibration period, the sample was exposed to the solvent by dropping the sample cartridge in the calorimeter cell. The resulting heat signal was measured by integrating the heat flow signal. Blank experiments were performed by dropping empty sample cartridges in the cell, and the related heat was subtracted from the dissolution heat signals. Experiments for each studied system were performed in duplicate or triplicate.

The acetonitrile (Fischer Chemicals, HPLC Gradient Grade) was used without further purification.

The oxalic acid dihydrate, **ox**•$2H_2O$ (Biomus) was dried for 4 hours at 110 °C and 1 hour in the desiccator over P_2O_5, in order to obtain anhydrous **ox** before weighing.

S4 Results of calculations and experiments

S4.1 Periodic DFT calculation results

Table S1 Periodic DFT electronic energies of individual cocrystal components.

Compound	CSD Refcode	Electronic energy per primitive unit cell / eV		Electronic energy per formula unit / eV	
		PBE+D3	PBE+MBD*	PBE+D3	PBE+MBD*
**2,2'-bipy**	BIPYRL04	-4517.100	-4517.516	-2258.550	-2258.758
**4,4'-bipy**	HIQWEJ02	-9033.731	-9034.481	-2258.433	-2258.620
**dabco**	TETDAM	-3385.259	-3385.763	-1692.630	-1692.88
**acr**	ACRDIN07	-19771.361	-19773.811	-2471.420	-2471.726
**tmpyr**	MPYRAZ01	-8036.767	-8037.599	-2009.192	-2009.400
**pyr**	PYRAZI01	-2495.474	-2495.584	-1247.737	-1247.792
**tftib**	UCEPEY	-21266.160	-21266.476	-5316.540	-5316.619
**1,4tfib**	ZZZAVM02	-10365.007	-10365.157	-5182.503	-5182.579
**fum**	FUMAAC	-14718.769	-14719.106	-2453.128	-2453.184
**pht**	PHTHAC01	-6232.679	-6232.982	-3116.339	-3116.491
**trdec***	TRDECA	-6702.763<br>-6702.684	-6703.507<br>-6703.435	-3351.362 (average)	-3351.736 (average)
**succ**	SUCACB02	-4972.933	-4973.048	-2486.467	-2486.524
**ox**	OXALAC07	-4211.026	-4211.095	-2105.513	-2105.548

* In experimental structure of **trdec** (CSD TRDECA) the position of the carboxylic group proton is not reported. Two optimizations were performed, placing the proton at each of the carboxylic group oxygen atoms. Then the average energy of the from the two calculations was computed.

Table S2. Periodic DFT electronic energies of cocrystals, and calculated formation energies from starting materials.

Cocrystal	CSD Refcode	Electronic energy per primitive unit cell / eV		Electronic energy per formula unit / eV		Cocrystal formation energy / kJ mol^{-1}	
		PBE+D3	PBE+MBD*	PBE+D3	PBE+MBD*	PBE+D3	PBE+MBD*
(**2,2’-bipy**)(**tftib**)	KIHREB	-30301.100	-30302.213	-7575.275	-7575.553	-17.88	-17.00
(4,**4’-bipy**)(**tftib**)	WEXWEC	-15150.627	-15151.150	-7575.314	-7575.575	-32.90	-32.38
(**dabco**)(**tftib**)	RORWIG	-28038.202	-28039.271	-7009.550	-7009.818	-30.59	-36.76
(**acr**)(**tftib**)	SAJDAL	-31153.908	-31152.457	-7788.477	-7788.114	-12.71	-14.89
(**tmpyr**)(**tftib**)	SAJCUE	-29303.861	-29304.833	-7325.965	-7326.208	-22.53	-18.27
(**pyr**)$_{1/2}$(**tftib**)	this work	-23762.234	-23762.570	-5940.559	-5940.642	-14.48	-12.30
(**pyr**)(14**tfib**)	WIGSUD	-12861.067	-12861.309	-6430.534	-6430.655	-28.28	-27.42
(**pyr**)(**fum**)	VAXVOH	-3701.023	-3701.126	-3701.023	-3701.126	-15.21	-14.47
(**pyr**)(**pht**)	VAXVUN	-17456.486	-17457.362	-4364.121	-4364.341	-4.34	-5.56
(**pyr**)(**trdec**)	IDAQEK	-7950.634	-7951.327	-7950.634	-7951.327	-16.75	-6.20
(**pyr**)(**succ**)	VAXWAU	-7468.704	-7468.916	-3734.352	-3734.458	-14.28	-13.71
(**pyr**)(**ox**)	GUDSUV	-13414.499	-13414.802	-3353.625	-3353.701	-36.14	-34.84

S4.2 Results of mechanochemical and slurry experiments

Table S3. Summary of Rietveld quantitative phase analysis for all mechanochemistry and slurry experiments. The percentages in the table refer to weight fractions.

System number	Reactants	Expected product from theoretical prediction	Liquid-assisted grinding			Slurry
			Ethanol	Acetonitrile	Hexane	
**1**	**(pyr)**$_{1/2}$**(tftib)** + **2,2'-bipy**	**(2,2'-bipy) (tftib)**	**(2,2'-bipy) (tftib)** = 100%			**(2,2'-bipy) (tftib)** = 100%
	**(2,2'-bipy) (tftib)** + ½ **pyr**		**(2,2'-bipy) (tftib)**= 100%	**(2,2'-bipy) (tftib)**= 100%	Just after grinding, **(2,2'-bipy) (tftib)** mixed with an unknown phase ref **(Figure S2)**<br><br>After one month **(2,2'-bipy) (tftib)** =100% ref **(Figure S3)**	
**2**	**(pyr)**$_{1/2}$**(tftib)** + **4,4'-bipy**	**(4,4'-bipy) (tftib)**	**(4,4'-bipy) (tftib)** = 100%			**(4,4'-bipy) (tftib)** = 100%
	**(4,4'-bipy) (tftib)** + ½ **pyr**		**(4,4'-bipy) (tftib)** = 100%	**(4,4'-bipy) (tftib)** = 100%	**(4,4'-bipy) (tftib)** = 100%	

Table S3. Continuation.

**3**	**(pyr)$_{1/2}$(tftib)** + **dabco**	**(dabco) (tftib)**	**(dabco) (tftib)**= 100%			**(dabco) (tftib)**= 21.87% (**dabco**)$_2$(**tftib**)= 78.13%
	**(dabco) (tftib)** + ½ **pyr**		**(dabco) (tftib)**= 100%	**(dabco) (tftib)**= 100%	Just after grinding, **(dabco) (tftib)** mixed with an unknown phase ref **(Figure S4)**<br><br>After two months<br>**(dabco) (tftib)**= 100%<br>ref **(Figure S5)**	
**4**	**(pyr)$_{1/2}$(tftib)** + **acr**	**(acr) (tftib)**	**(acr)(tftib)** = 100%			**(acr)(tftib)** = 100%
	**(acr) (tftib)** + ½ **pyr**		**(acr)(tftib)** = 100%	**(acr)(tftib)** = 100%	Just after grinding, **(acr)(tftib)** = 65.85%; **(pyr)$_{1/2}$(tftib)**= 34.15% ref **(Figure S6)**<br><br>After three months<br>**(acr)(tftib)** = 70.40 %<br>**(pyr)$_{1/2}$(tftib)**= 13.10 %<br>**tftib** = 16.50 %<br>ref **(Figure S7)**	
**5**	**(pyr)$_{1/2}$(tftib)** + **tmpyr**	**(tmpyr) (tftib)**	**(tmpyr)(tftib)** = 100%			**(tmpy)(tftib)** = 100%
	**(tmpyr) (tftib)** + ½ **pyr**		**(tmpyr)(tftib)** = 100%	**(tmpyr)(tftib)** = 100%	**(tmpyr)(tftib)** = 100%	

Table S3. Continuation.

**6**	**(pyr)$_{1/2}$(tftib)** + ½ **14tfib**	**(pyr)$_{1/2}$(tftib)**	**(pyr)$_{1/2}$(tftib)**= 42.09% **1,4tfib** = 57.91%	**(pyr)$_{1/2}$(tftib)**= 62.88% **1,4tfib** = 37.12%	**(pyr)$_{1/2}$(tftib)**= 62.69% **1,4tfib** = 37.31%	**(pyr)$_{1/2}$(tftib)** = 82.34% **1,4tfib** = 17.66%
	½ **(pyr)(14tfib)** + **tftib**		**(pyr)$_{1/2}$(tftib)** = 70.87% **1,4tfib** = 29.13%			
**7**	**(pyr)$_{1/2}$(tftib)** + ½ **fum**	**(pyr)$_{1/2}$(tftib)**	**(pyr)$_{1/2}$(tftib)** = 78.65% **fum** = 21.35%	**(pyr)$_{1/2}$(tftib)** = 92.15% **fum** = 7.49%	**(pyr)$_{1/2}$(tftib)** = 77.50% **fum** = 22.50%	**(pyr)$_{1/2}$(tftib)** = 80.33% **fum** = 19.67%
	½ **(pyr)(fum)** + **tftib**		**(pyr)$_{1/2}$(tftib)** = 96.34% **fum** = 3.66%			
**8**	**(pyr)$_{1/2}$(tftib)** + ½ **pht**	**(pyr)$_{1/2}$(tftib)**	**(pyr)$_{1/2}$(tftib)** = 61.30% **pht** = 38.70%	**(pyr)$_{1/2}$(tftib)** = 85.27% **pht** = 14.73%	**(pyr)$_{1/2}$(tftib)** = 83.10% **pht** = 16.90%	**(pyr)$_{1/2}$(tftib)** = 98.08% **pht** = 1.92%
	½ **(pyr)(pht)** + **tftib**		**(pyr)$_{1/2}$(tftib)** = 75.34% **pht** = 24.66%			

Table S3. Continuation.

**9**	**(pyr)$_{1/2}$(tftib)** + **tdec**	**(pyr)$_{1/2}$(tftib)**	**(pyr)$_{1/2}$(tftib) =** 82.41 %<br>**tdec - - TRDECA =** 5.38 %<br>**tdec- TRDECA01 - =** 12.21 %	**(pyr)$_{1/2}$(tftib) =** 77.02 %<br>**tdec - TRDECA =** 5.23 %<br>**tdec- TRDECA01 =** 17.75 %	Just after grinding, **(pyr)$_{1/2}$(tftib) = 81.19** %;<br>**(pyr)(tdec)** = 18.81 % ref **(Figure S8)**<br><br>After two months<br>**(pyr)$_{1/2}$(tftib) =** 60.09 %<br>**tdec- TRDECA =** 20.49 %<br>**tdec- TRDECA01 =** 19.42 %<br>ref **(Figure S9)**	**(pyr)$_{1/2}$(tftib) =** 54.99 %<br>**tdec- TRDECA =** 45.01 %
	½ **(pyr) (tdec)$_2$** + **tftib**		**½ hr grinding**<br>**(pyr)$_{1/2}$(tftib) =** 49.98%<br>**(pyr) (tdec)$_2$** = 2.71%<br>**tdec- TRDECA01 =** 47.30%<br>**1hr grinidng**<br>**(pyr)$_{1/2}$(tftib) =** 55.84 %<br>**tdec - TRDECA =** 2.65 %<br>**tdec- TRDECA01 =** 12.18**%**<br>**tftib** = 29.33%			

Table S3. Continuation.

**10**	**(pyr)**$_{1/2}$**(tftib)** + **succ**	**(pyr)**$_{1/2}$**(tftib)**	**(pyr)**$_{1/2}$**(tftib)** =<br>90.32%<br>**succ** = 9.68%	**(pyr)**$_{1/2}$**(tftib)** =<br>97.22%<br>**succ** = 2.78%	Just after grinding, **(pyr)**$_{1/2}$**(tftib)** = 83.26%;<br>**(succ)(pyr)**= 16.74% ref **(Figure S10)**<br><br>After three months<br>**(pyr)**$_{1/2}$**(tftib) =** 89.60%<br>**succ=** 10.40%<br>ref **(Figure S11)**	**(pyr)**$_{1/2}$**(tftib)** =<br>82.05%<br>**(succ)(pyr) =**13.86%<br>**succ** = 4.09%
	**(succ)(pyr)** + **tftib**		**(pyr)**$_{1/2}$**(tftib)** =<br>93.26%<br>**succ** = 6.74%			
**11**	**(pyr)**$_{1/2}$**(tftib)** +**ox**	**(ox)(pyr)**	½ hr- **(ox)(pyr)** =<br>7.47%%<br>**(pyr)**$_{1/2}$**(tftib)** =<br>55.39%<br>tftib = 37.14%<br>1hr – **(ox)(pyr)** –<br>18.45%<br>**tftib**- 79.87%<br>**ox**•2H_2O - 1.68%			**(ox)(pyr)**= 25.86%<br>**(tftib)**= 74.14%
	**(ox)(pyr)** + **tftib**		**(ox)(pyr)**= 4.88%<br>**tftib** =95.12%	**(ox)(pyr)**= 6.00%<br>**tftib)** = 94.00%	**(ox)(pyr)**= 6.36%<br>**tftib)** = 93.64%	

S4.3 Rietveld refinements of reaction products

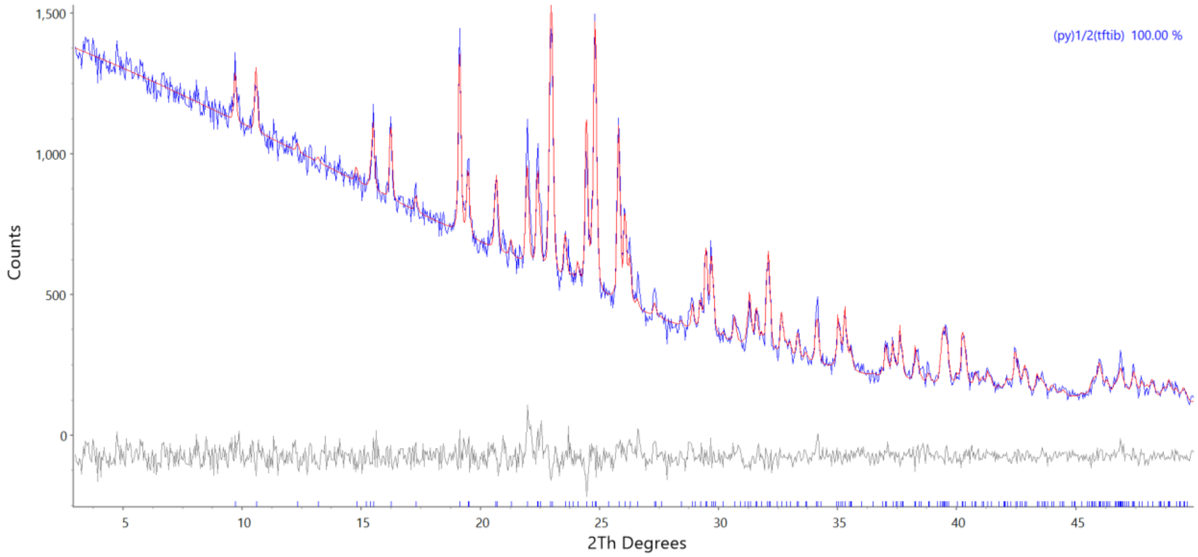

Figure S1. Rietveld refinement of the **(pyr)**$_{1/2}$**(tftib)** cocrystal. The experimental profile is shown in blue, calculated profile in red, and the difference curve in grey.

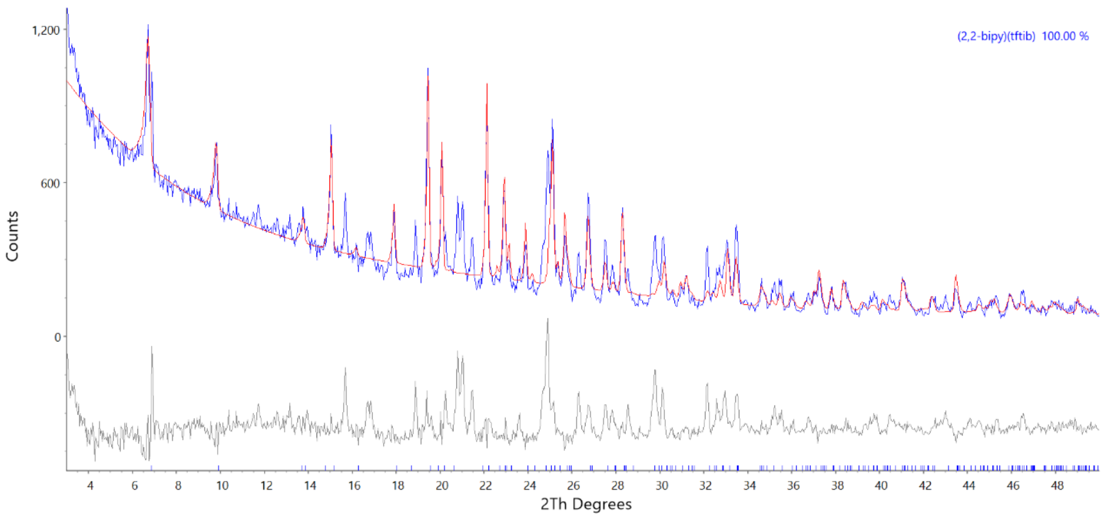

Figure S2. Rietveld refinement of the product mixture after the milling reaction of **(2,2'-bipy) (tftib)** + ½ **pyr** in hexane (Reaction 1, reverse), analyzed just after grinding. Formation of **(2,2'-bipy) (tftib)** cocrystal together with a solvated impurity of unknown crystal structure is seen. The solvated nature of the impurity was confirmed by TGA (**Figure S13**). Upon storing this sample for a month at RT, this solvated intermediate converted to the **(2,2'-bipy)(tftib)** cocrystal (**Figure S3)**. The experimental profile is shown in blue, calculated profile in red, and the difference curve in grey.

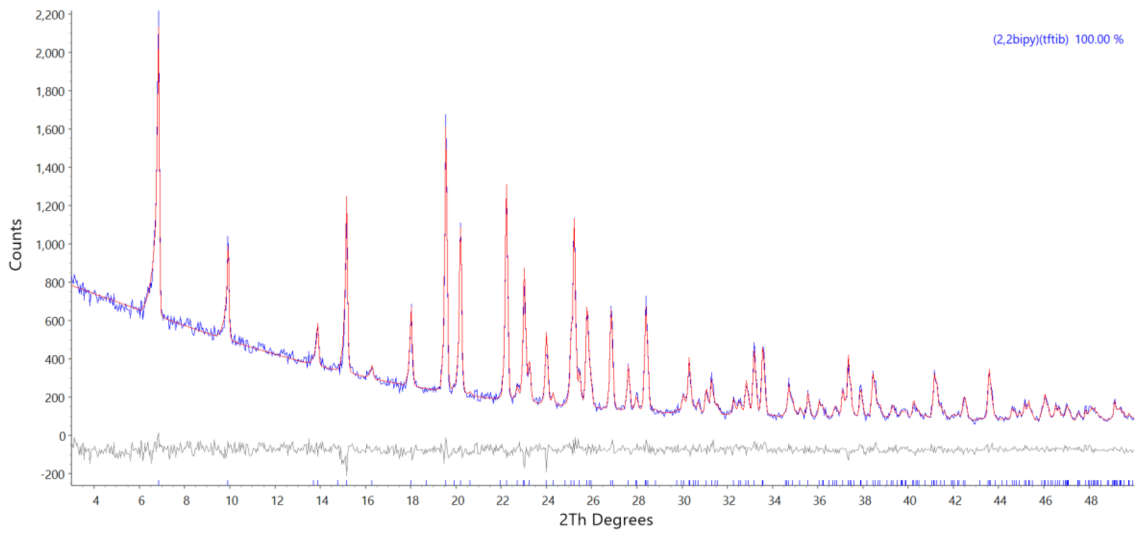

Figure S3. Rietveld refinement of the product of the milling reaction of **(2,2'-bipy) (tftib)** + ½ **pyr** cocrystal in hexane (Reaction 1, reverse), shows quantitative formation of **(2,2'-bipy)(tftib)** after a month of storing this sample at RT. The experimental profile is shown in blue, calculated profile in red, and the difference curve in grey.

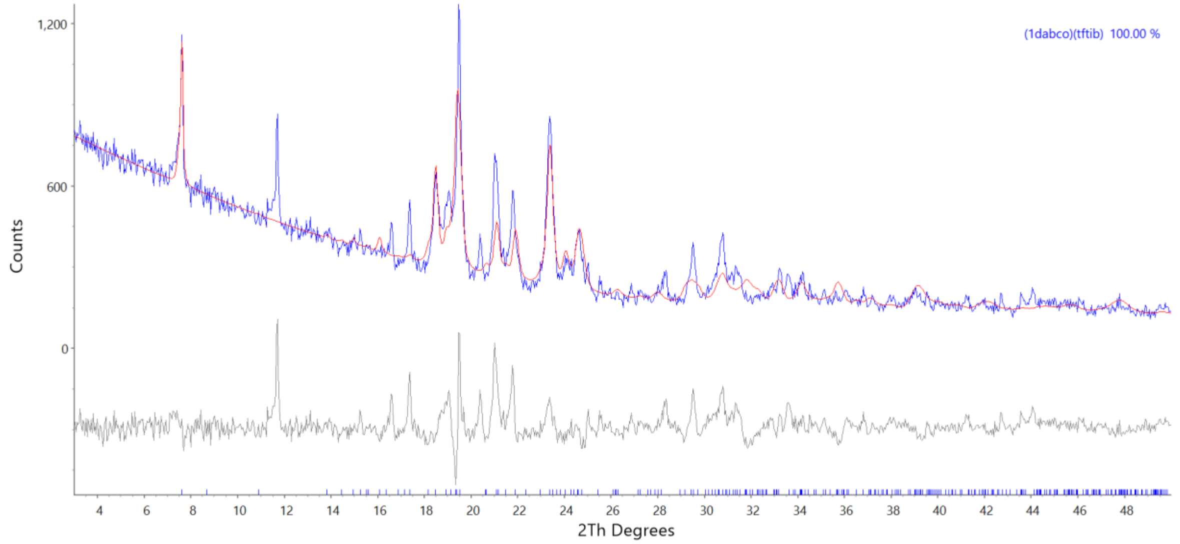

Figure S4. Rietveld refinement of the products of the **(dabco)(tftib)** + ½ **pyr** milling reaction in hexane (Reaction 3, reverse) just after grinding, showing the formation of **(dabco)(tftib)** cocrystal with a solvated impurity of unknown crystal structure. The impurity is identified as a solvate based on the TGA measurement (Figure S15). Upon storing this sample for two months at RT, this solvated intermediate fully converted to the unsolvated **(dabco)(tftib)** product **(Figure S5)**. The experimental profile is shown in blue, calculated profile in red, and the difference curve in grey.

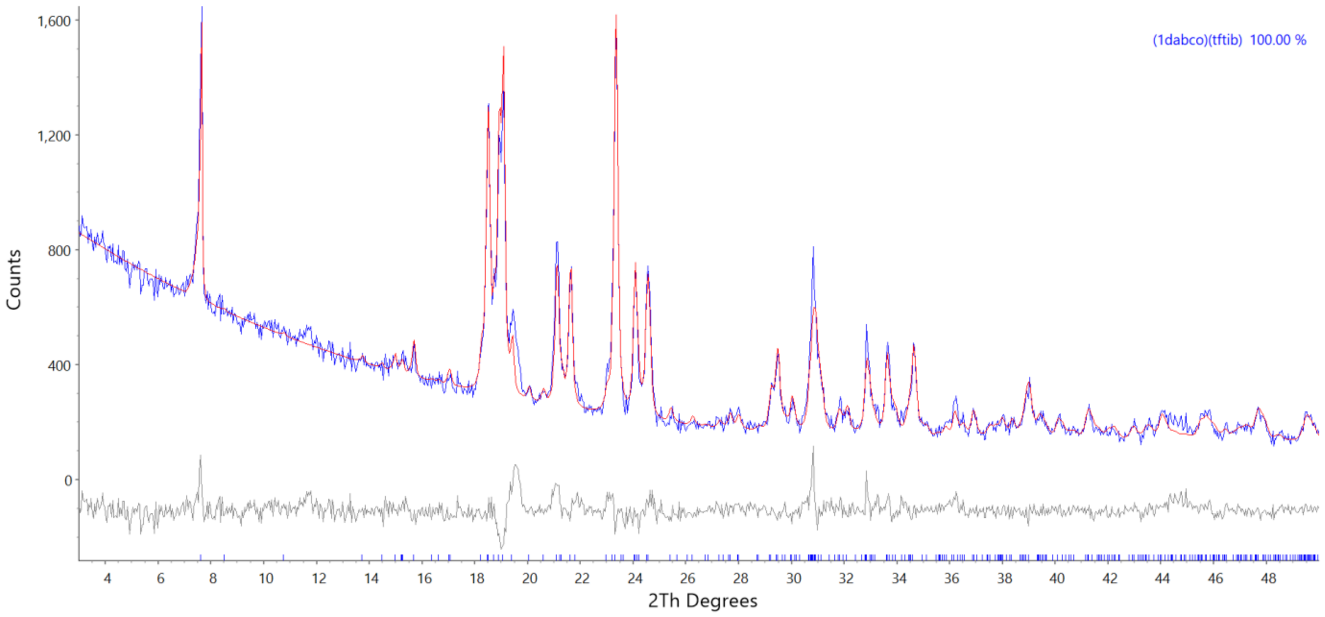

Figure S5. Rietveld refinement of the products of the **(dabco)(tftib)** + ½ **pyr** milling reaction in hexane (Reaction 3, reverse), shows quantitative formation of **(dabco) (tftib)** after two months of storing this sample at RT. The experimental profile is shown in blue, calculated profile in red, and the difference curve in grey.

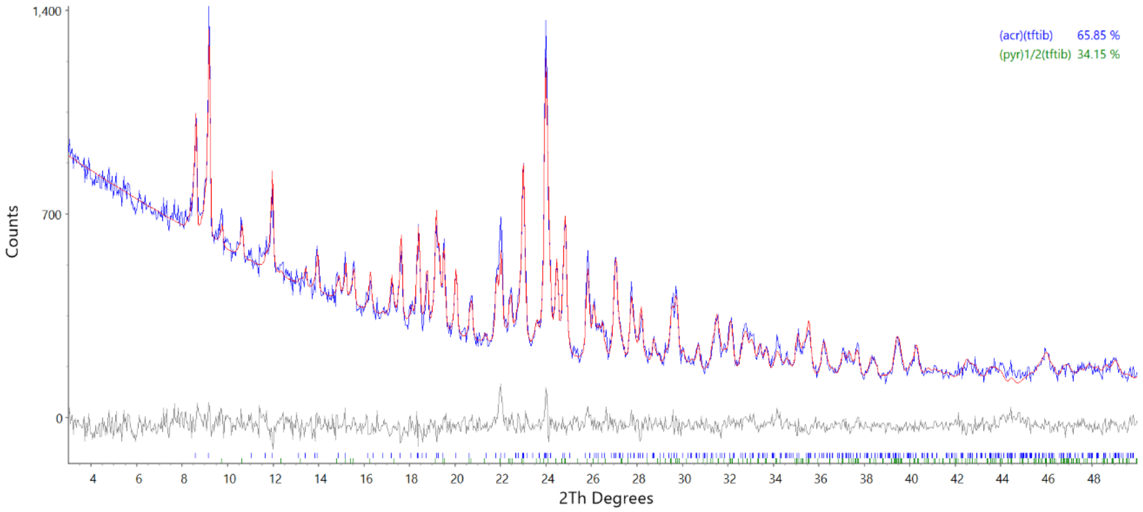

Figure S6. Rietveld refinement of **(acr) (tftib)** + ½ **pyr** reaction products after milling in hexane (Reaction 4, reverse), collected immediately after grinding. The product mixture contains **(acr) (tftib)** and **(pyr)**$_{1/2}$**(tftib)** cocrystals. Upon storing this sample for three months at RT, full conversion back to **(acr) (tftib)** cocrystal is observed (**Figure S7**). The experimental profile is shown in blue, calculated profile in red, and the difference curve in grey.

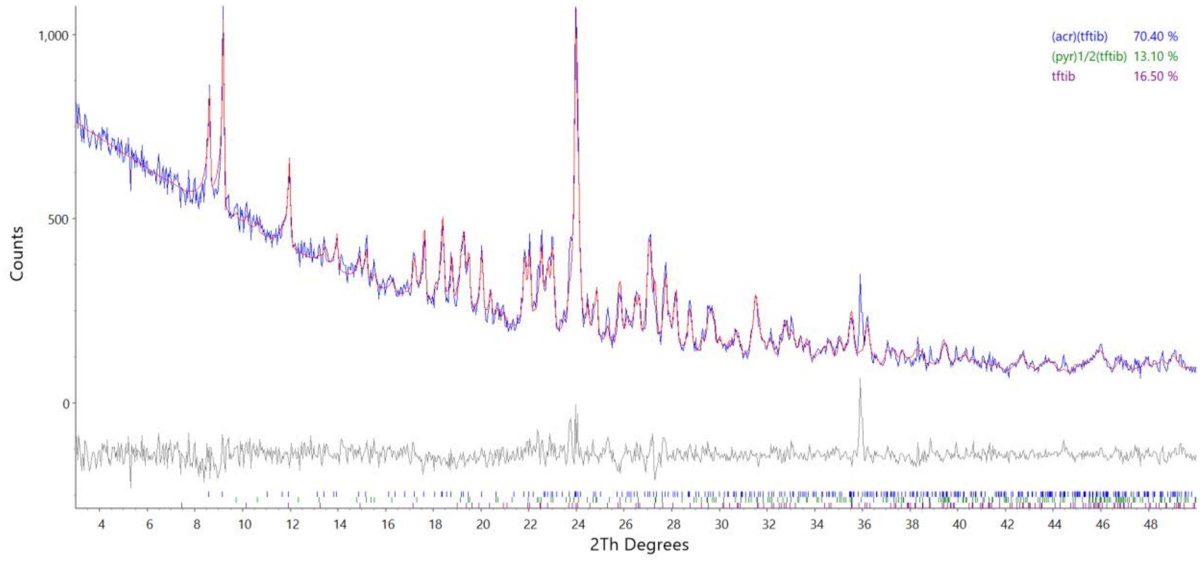

Figure S7. Rietveld refinement of **(acr) (tftib)** + ½ **pyr** reaction products after milling in hexane (Reaction 4, reverse), and storing the sample for three months at RT. Compared to the mixture composition immediately after milling (Figure S6) the mixture after storage contains more **(acr)(tftib)** cocrystal and less (**pyr**)$_{1/2}$(**tftib**), with an additional formation of crystalline **tftib**. The experimental profile is shown in blue, calculated profile in red, and the difference curve in grey.

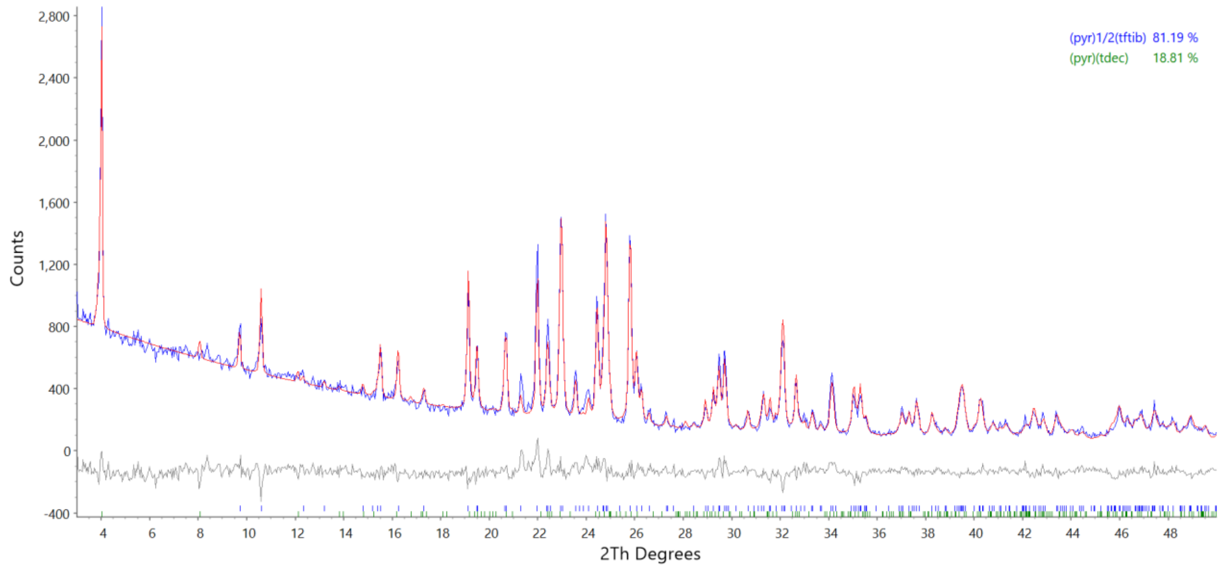

Figure S8. Rietveld refinement of **(pyr)$_{1/2}$(tftib)** + **tdec** cocrystal in hexane (Reaction 9, forward) just after grinding showing the formation of mixture of (**pyr)$_{1/2}$(tftib) and (pyr)(tdec)** cocrystals. Upon storing this sample for two months at RT, this mixture got converted to the thermodynamically favored product (**Figure S9)**. The experimental profile is shown in blue, calculated profile in red, and the difference curve in grey.

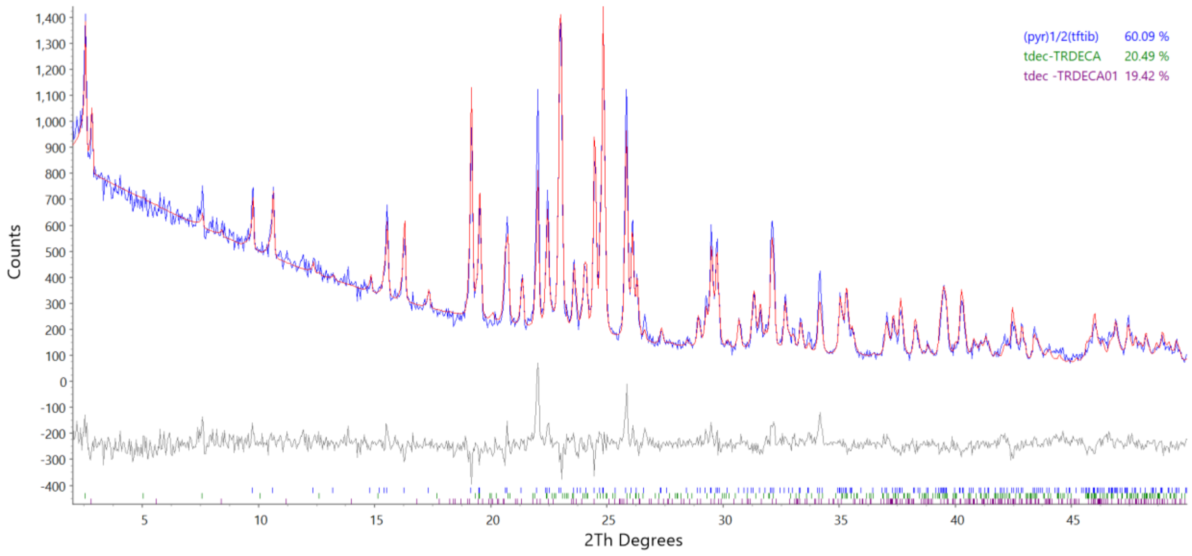

Figure S9. Rietveld refinement of **(pyr)$_{1/2}$(tftib)** + **tdec** cocrystal in hexane (Reaction 9, forward), shows the conversion to **(pyr)$_{1/2}$(tftib)** and two polymorphs of **tdec** after two months of storing the sample at RT. **tdec** reagent that has been used in this reaction is **P-1** (**TRDECA**), whereas the product obtained out of this reaction is two different polymorphs having **P-1** (**TRDECA**) and **C2/c** (**TRDECA01**) space groups. The experimental profile is shown in blue, calculated profile in red, and the difference curve in grey.

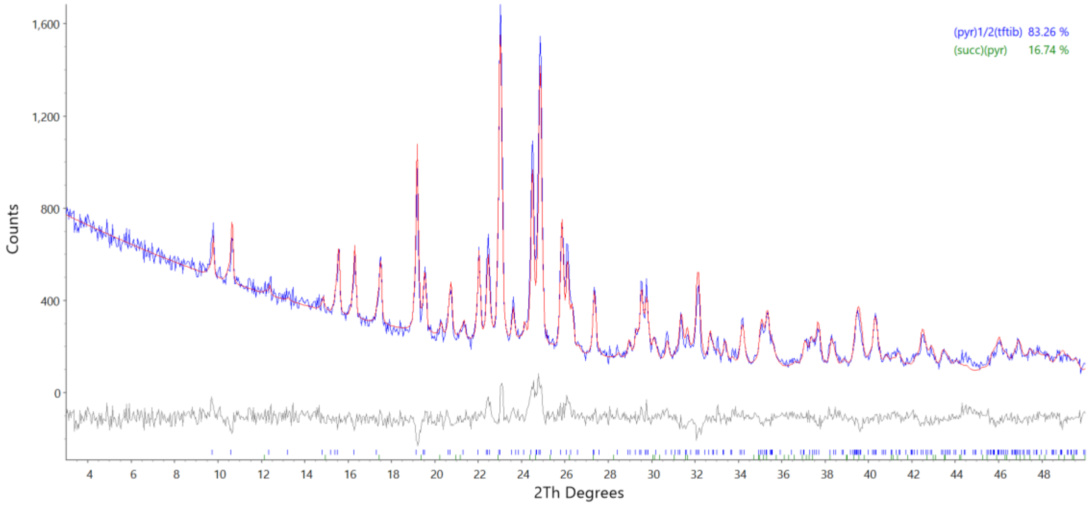

Figure S10. Rietveld refinement of the products of the milling reaction of **(pyr)$_{1/2}$(tftib)** + **succ** performed in hexane (Reaction 10, forward), with PXRD collected straight after grinding, shows the formation of a mixture of **(pyr)$_{1/2}$(tftib)** and **(pyr)(succ)** cocrystals. Upon storing this sample for three months at RT, this mixture got converted to the thermodynamically favored product (**Figure S11**). The experimental profile is shown in blue, calculated profile in red, and the difference curve in grey.

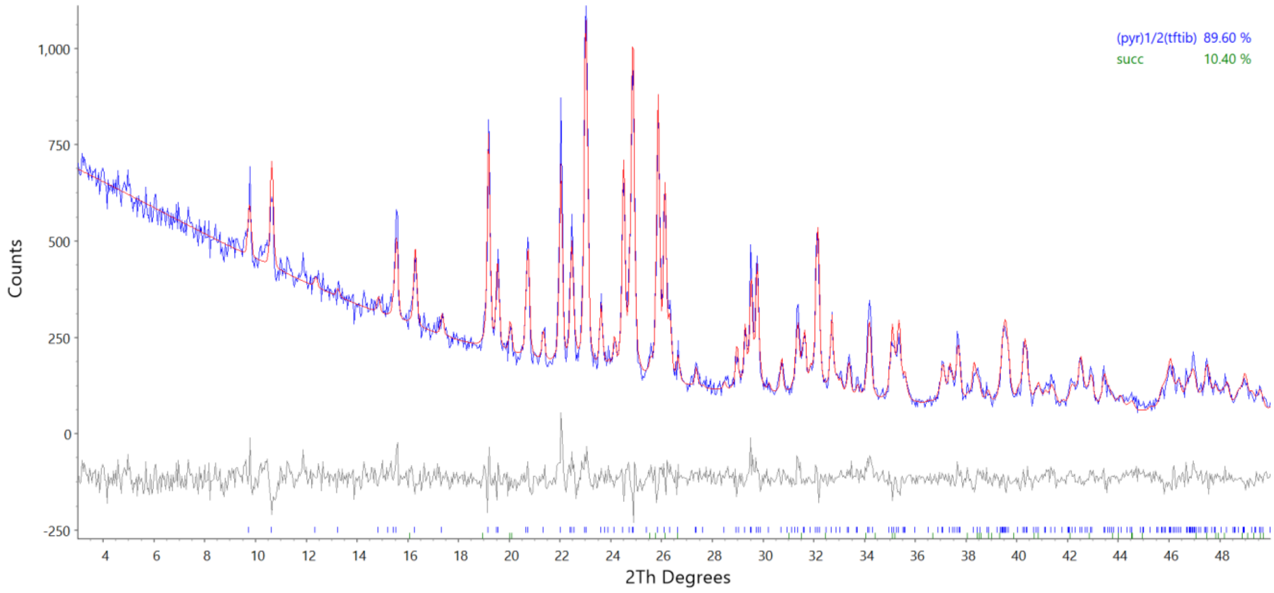

Figure S11. Rietveld refinement of the products of the milling reaction **(pyr)**$_{1/2}$**(tftib)** + **succ** performed in hexane (Reaction 10, forward), after storing the product at room temperature for three months. Quantitative formation of **(pyr)**$_{1/2}$**(tftib)** is observed. The experimental profile is shown in blue, calculated profile in red, and the difference curve in grey.

S4.3 Thermal measurements

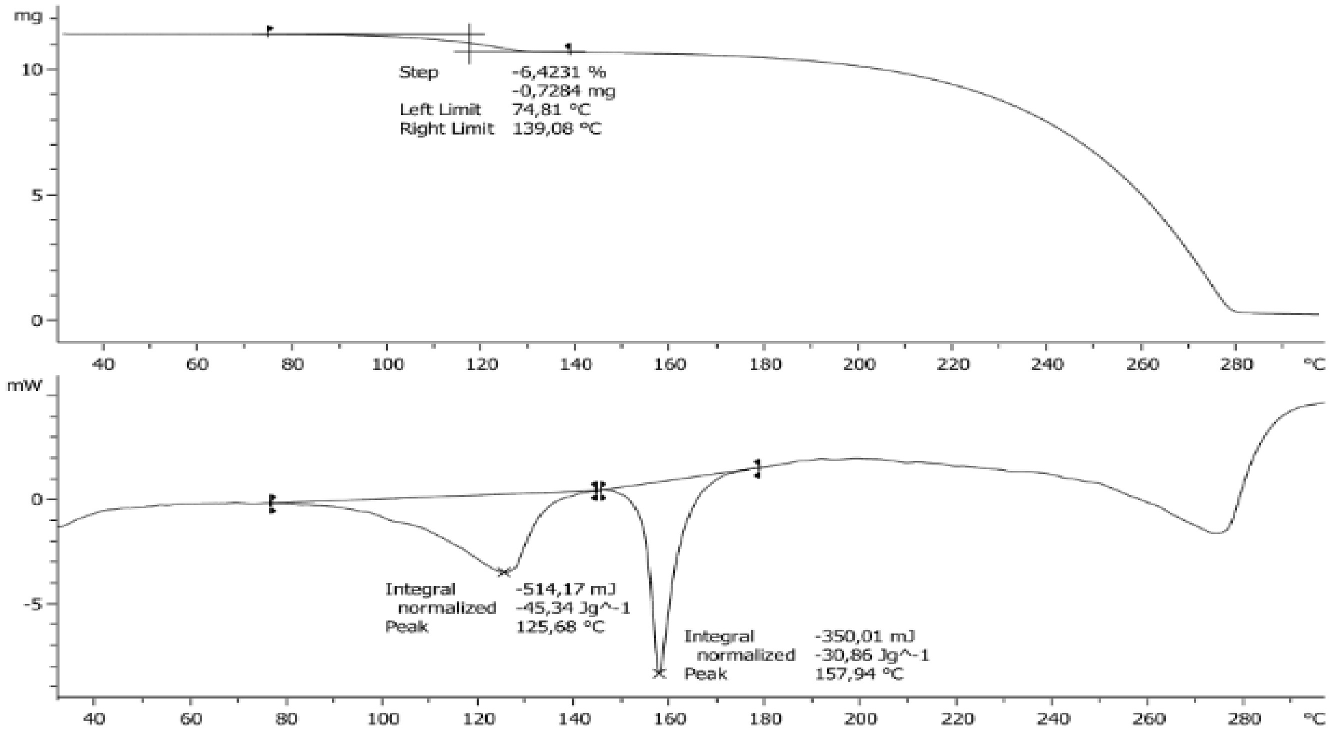

Figure S12. TGA/DSC curves recorded for the (**pyr**)$_{1/2}$(**tftib**) cocrystal. The observed weight loss at 70−130 ºC, is consistent with the amount of pyrazine within the investigated cocrystal (experimental weight loss 6.42%, calculated weight loss 7.28%). This weight loss is accompanied by a broad endothermic peak at 125.68 °C in the DSC curve.

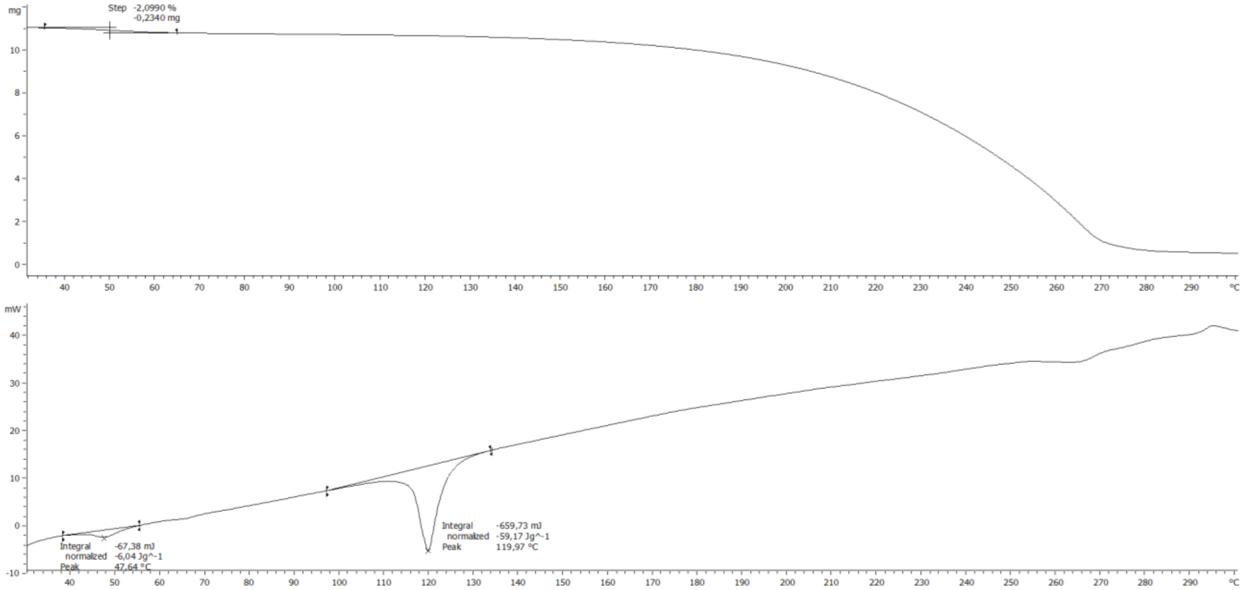

Figure S13. TGA/DSC curves recorded for the product obtained from milling (**2,2'-bipy**) (**tftib**) + ½ **pyr** in hexane. Low temperature weight loss shows the presence of solvated impurity just after grinding. Above the solvent loss temperature the DSC trace is consistent with that of the pure (**2,2'-bipy**)(**tftib**) cocrystal (Figure S14).

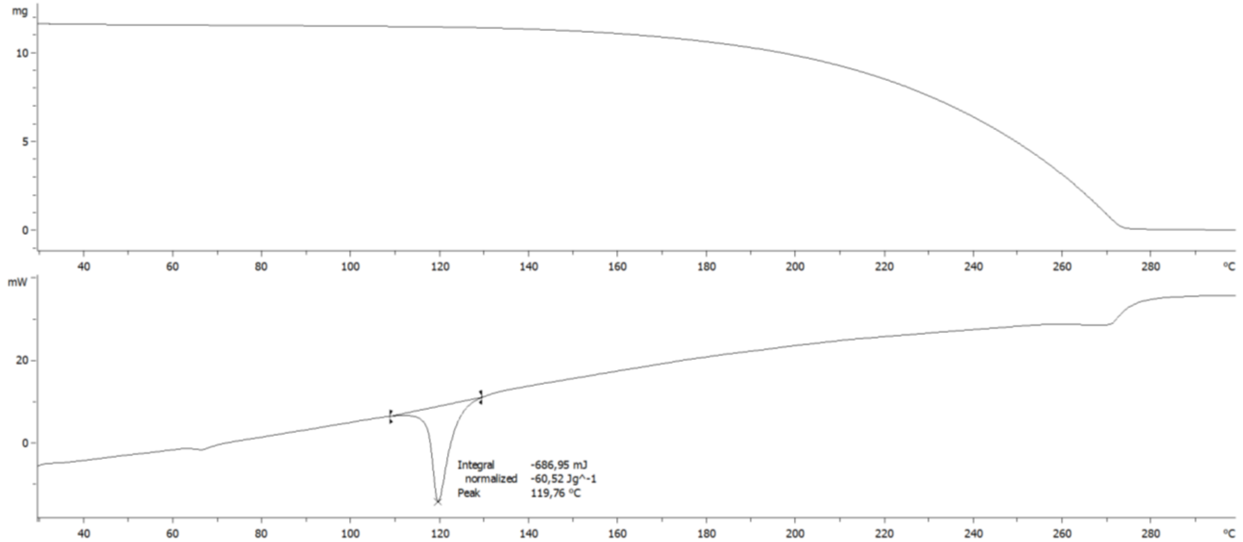

Figure S14. TGA/DSC curves recorded for (**2,2'-bipy**)(**tftib**) cocrystal. No solvent-related weight loss is observed. The endothermic event with a peak at 119.76 °C corresponds to the melting of (**2,2'-bipy**)(**tftib**) cocrystal

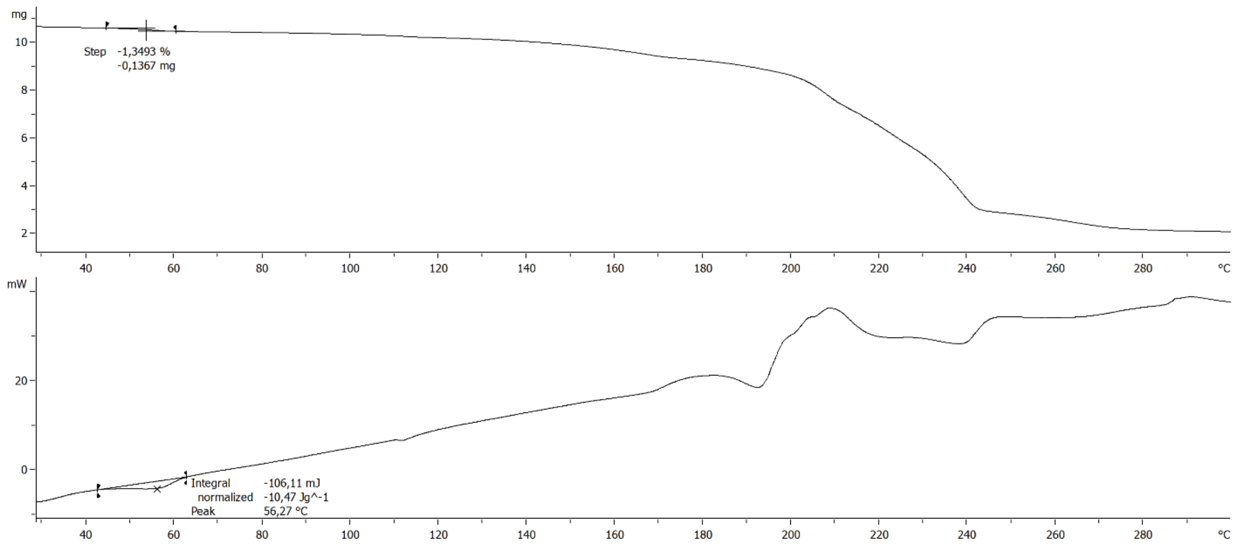

Figure S15. TGA/DSC curves recorded for product obtained from milling (**dabco**) (**tftib**) + ½ **pyr** in hexane. Low temperature weight loss and DSC endothermic event with a peak at 56.27 °C indicate the presence of solvated impurity just after grinding. Above the solvent loss temperature the DSC trace is consistent with that of the pure (**dabco**) (**tftib**) cocrystal (Figure S16).

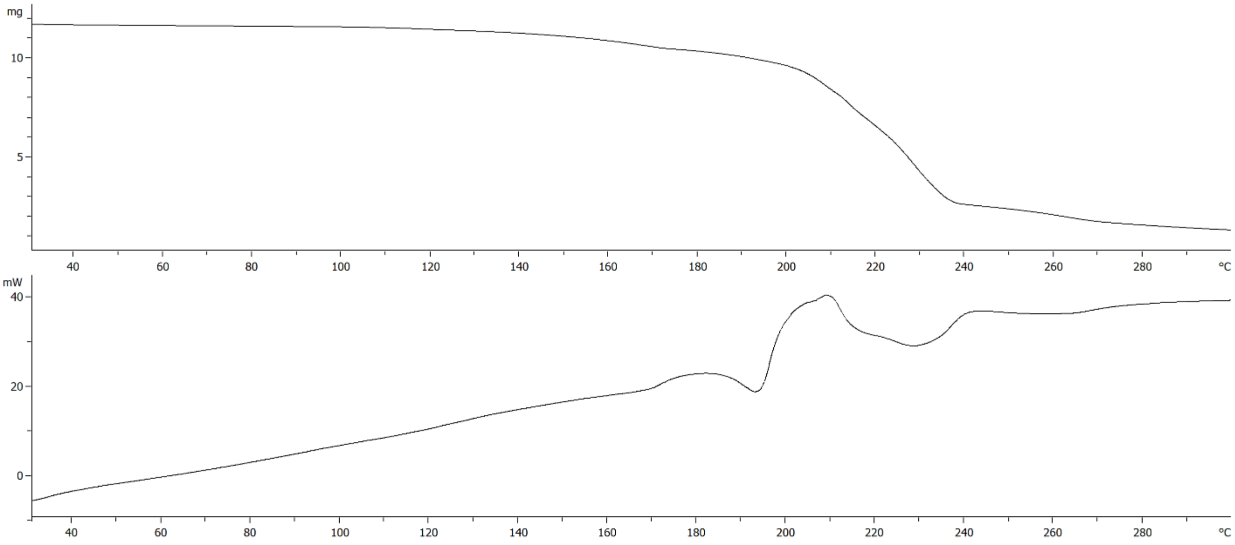

Figure S16. TGA/DSC curves recorded for (**dabco**) (**tftib**) cocrystal.

S4.4 Crystallographic information

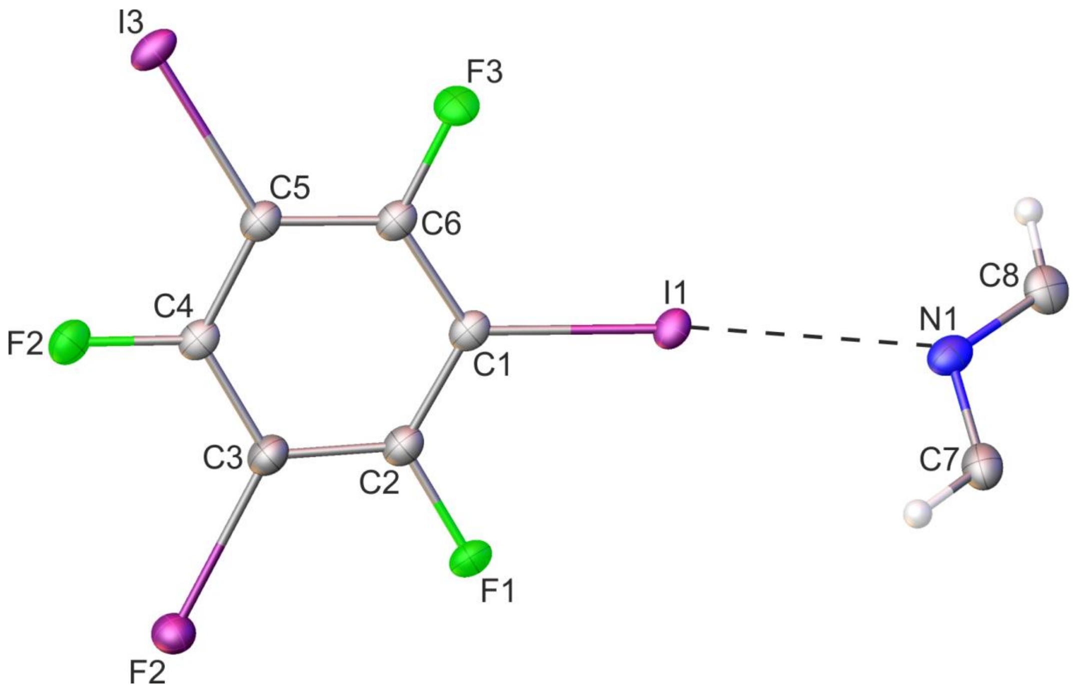

Figure S17. Asymmetric unit of the crystal lattice of **(pyr)$_{1/2}$(tftib)** with atom labelling scheme. Displacement ellipsoids are drawn at the 50% probability level and the H-atoms are shown as small spheres of arbitrary radius. The N···I halogen bond is represented by a dashed line.

Table S4. Crystal data and structure refinement details for **(pyr)$_{1/2}$(tftib)**.

Empirical formula	$C_8H_2F_3I_3N$
CCDC Number	2217175
Formula weight	549.81
Temperature/K	100(2)
Crystal system	monoclinic
Space group	$P2_1/c$
a/Å	9.1685(4)
b/Å	7.7161(3)
c/Å	16.7661(6)
α/°	90
β/°	99.439(4)
γ/°	90
Volume/Å^3	1170.06(8)
Z	4
ρ_{calc}g/cm^3	3.121
μ/mm^1	8.019
F(000)	972.0
Crystal size/mm^3	0.71 × 0.61 × 0.44
Radiation	Mo$K\alpha$ (λ = 0.71073)
2Θ range for data collection/°	4.504 to 50.246
Index ranges	$-10 \leq h \leq 10$, $-9 \leq k \leq 9$, $-20 \leq l \leq 20$
Reflections collected	13981
Independent reflections	2080 [R_{int} = 0.1146, R_{sigma} = 0.0570]
Data/restraints/parameters	2080/48/137
Goodness-of-fit on F^2	1.159
Final R indexes [I>=2σ (I)]	R_1 = 0.0463, wR_2 = 0.1135
Final R indexes [all data]	R_1 = 0.0484, wR_2 = 0.1156
Largest diff. peak/hole / e Å^{-3}	1.89/-1.53

Table S5. Bond lengths for **(pyr)$_{1/2}$(tftib)**.

Atom	Atom	Length/Å	Atom	Atom	Length/Å
C1	C2	1.377(12)	C4	F2	1.336(9)
C1	C6	1.368(12)	C5	C6	1.400(11)
C1	I1	2.087(8)	C5	I3	2.086(8)
C2	C3	1.395(12)	C6	F3	1.343(9)
C2	F1	1.355(10)	C7	C8[1]	1.396(13)
C3	C4	1.388(12)	C7	N1	1.338(12)
C3	I2	2.086(8)	C8	C7[1]	1.396(13)
C4	C5	1.390(12)	C8	N1	1.337(11)

[1]2-X,-Y,1-Z

Table S6. Values of valence angles for **(pyr)$_{1/2}$(tftib)**.

Atom	Atom	Atom	Angle/°	Atom	Atom	Atom	Angle/°
C2	C1	I1	120.7(6)	F2	C4	C5	118.9(7)
C6	C1	C2	116.7(8)	C4	C5	C6	118.0(8)
C6	C1	I1	122.6(6)	C4	C5	I3	120.7(6)
C1	C2	C3	124.2(8)	C6	C5	I3	121.3(6)
F1	C2	C1	119.2(7)	C1	C6	C5	122.7(8)
F1	C2	C3	116.6(7)	F3	C6	C1	119.1(7)
C2	C3	I2	122.1(6)	F3	C6	C5	118.1(7)
C4	C3	C2	116.6(8)	N1	C7	C8[1]	122.7(8)
C4	C3	I2	121.3(6)	N1	C8	C7[1]	120.6(8)
C3	C4	C5	121.7(7)	C8	N1	C7	116.6(8)
F2	C4	C3	119.4(7)				

[1]2-X,-Y,1-Z

Table S7. Values of torsion angles for **(pyr)$_{1/2}$(tftib)**.

A	B	C	D	Angle/°	A	B	C	D	Angle/°
C1	C2	C3	C4	0.8(13)	F1	C2	C3	I2	1.1(11)
C1	C2	C3	I2	-177.6(6)	F2	C4	C5	C6	-179.4(7)
C2	C1	C6	C5	-2.6(12)	F2	C4	C5	I3	1.3(10)
C2	C1	C6	F3	180.0(7)	I1	C1	C2	C3	-179.0(7)
C2	C3	C4	C5	-1.8(12)	I1	C1	C2	F1	2.3(10)
C2	C3	C4	F2	178.2(7)	I1	C1	C6	C5	177.8(6)
C3	C4	C5	C6	0.6(12)	I1	C1	C6	F3	0.3(11)
C3	C4	C5	I3	-178.7(6)	I2	C3	C4	C5	176.6(6)
C4	C5	C6	C1	1.7(12)	I2	C3	C4	F2	-3.4(11)
C4	C5	C6	F3	179.1(7)	I3	C5	C6	C1	-179.0(6)
C6	C1	C2	C3	1.4(13)	I3	C5	C6	F3	-1.6(10)
C6	C1	C2	F1	-177.4(7)	C7[1]	C8	N1	C7	0.6(13)
F1	C2	C3	C4	179.5(7)	C8[1]	C7	N1	C8	-0.7(13)

[1]2-X,-Y,1-Z

S4.5 Dissolution calorimetry measurements

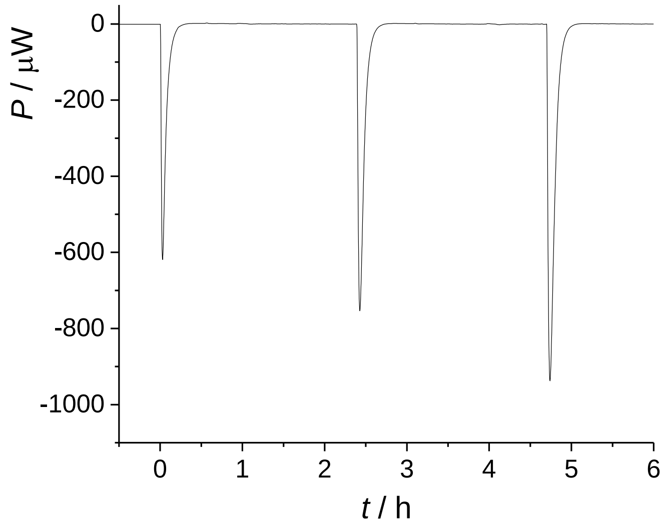

Figure S18. Calorimetric determination of dissolution enthalpy of **tftib** (m_1=4.71 mg, m_2=7.15 mg, m_3=9.84 mg) in acetonitrile at 25°C; stirring rate = 60 rpm.

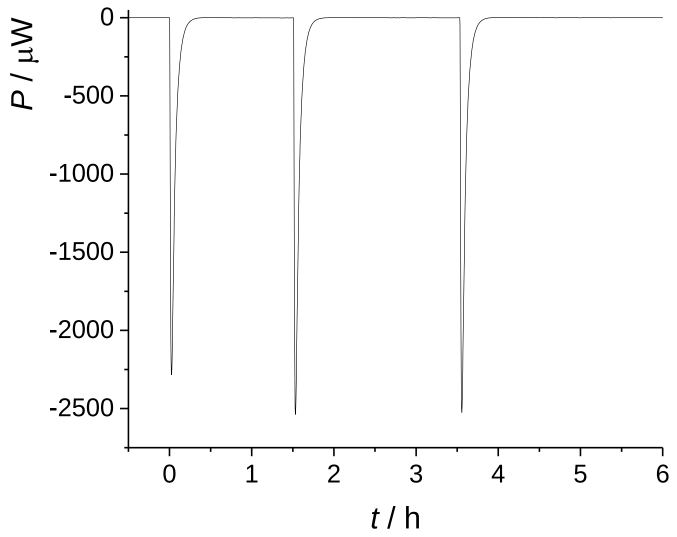

Figure S19. Calorimetric determination of dissolution enthalpy of **2,2'-bipy** (m_1=3.93 mg, m_2=4.61 mg, m_3=4.37 mg) in acetonitrile at 25°C; stirring rate = 60 rpm.

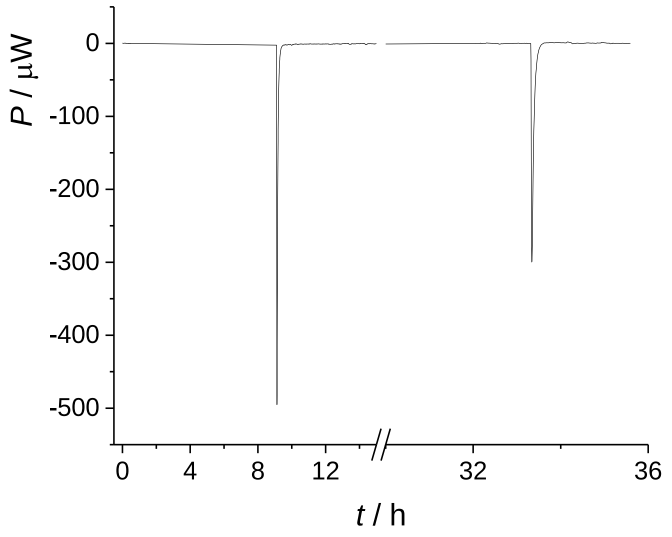

Figure S20. Calorimetric determination of dissolution enthalpy of (**2,2'-bipy**)(**tftib**) (m_1=1.640 mg, m_2=1.035 mg) in acetonitrile at 25°C; stirring rate = 60 rpm.

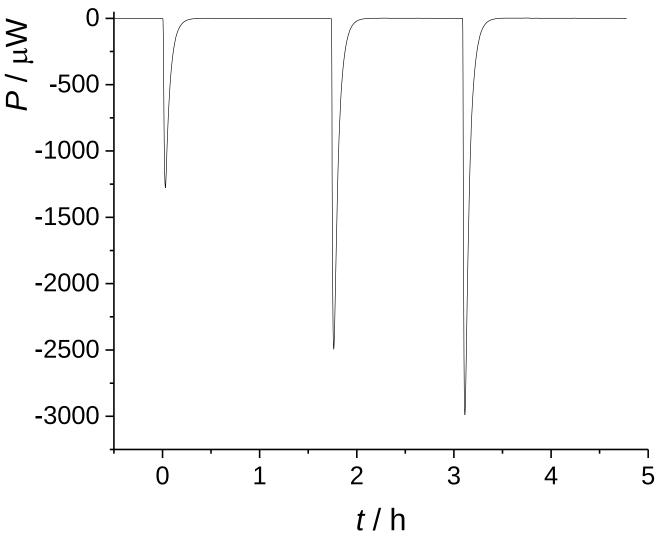

Figure S21. Calorimetric determination of dissolution enthalpy of **4,4'-bipy** (m_1=2.58 mg, m_2=5.10 mg, m_3=5.80 mg) in acetonitrile at 25°C; stirring rate = 60 rpm.

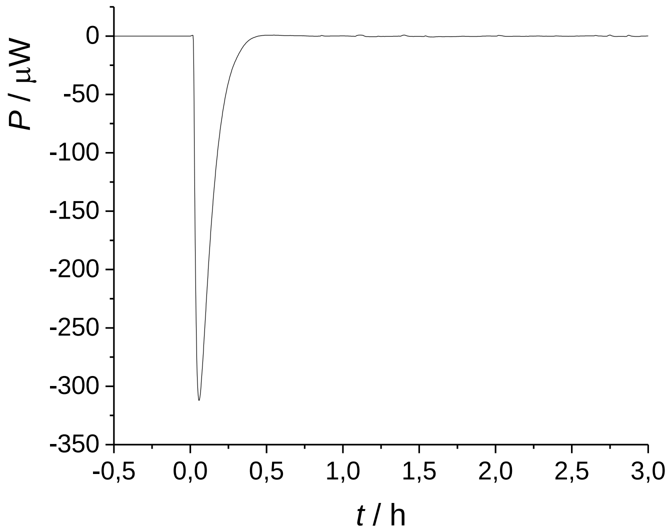

Figure S22. Calorimetric determination of dissolution enthalpy of (**4,4’-bipy**)(**tftib**) (*m*=1.755 mg) in acetonitrile at 25°C; stirring rate = 60 rpm.

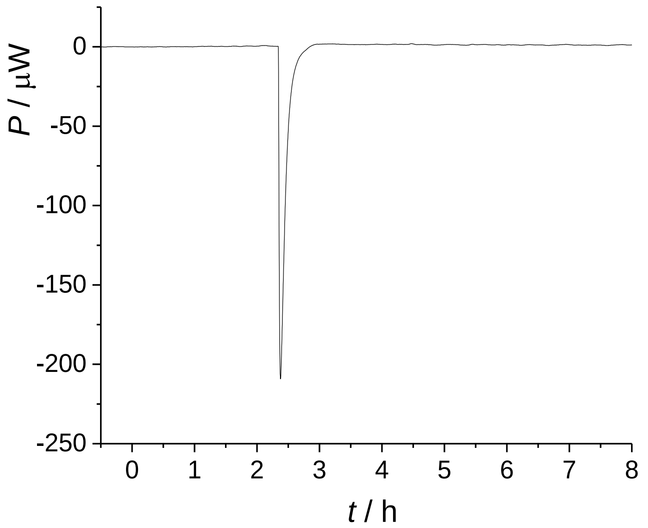

Figure S23. Calorimetric determination of dissolution enthalpy of (**4,4’-bipy**)(**tftib**) (*m*=1.130 mg) in acetonitrile at 25°C; stirring rate = 60 rpm.

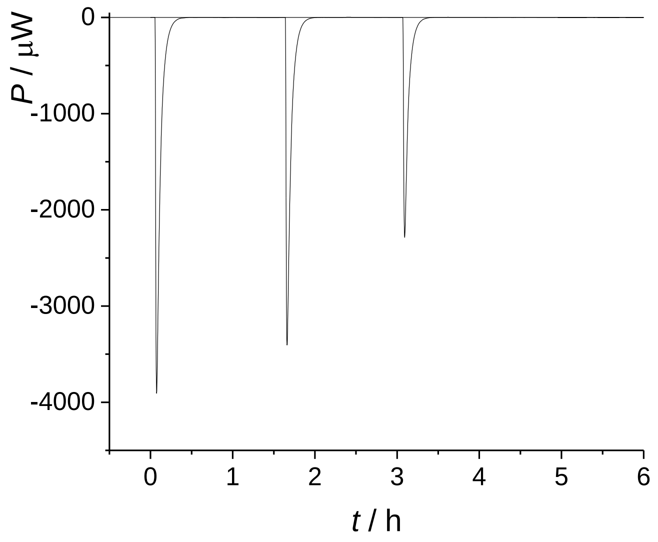

Figure S24. Calorimetric determination of dissolution enthalpy of **dabco** (m_1=5.23 mg, m_2=5.40 mg, m_3=3.01 mg) in acetonitrile at 25°C; stirring rate = 60 rpm.

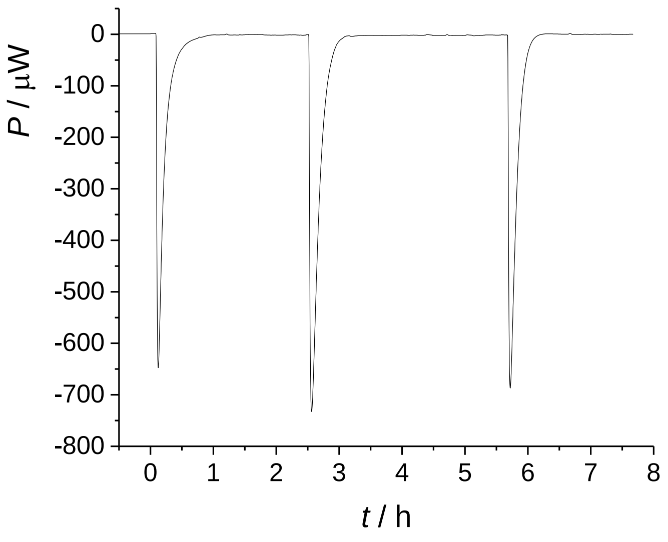

Figure S25. Calorimetric determination of dissolution enthalpy of (**dabco**)(**tftib**) (m_1=3.79 mg, m_2=5.02 mg, m_3=4.02 mg) in acetonitrile at 25°C; stirring rate = 60 rpm.

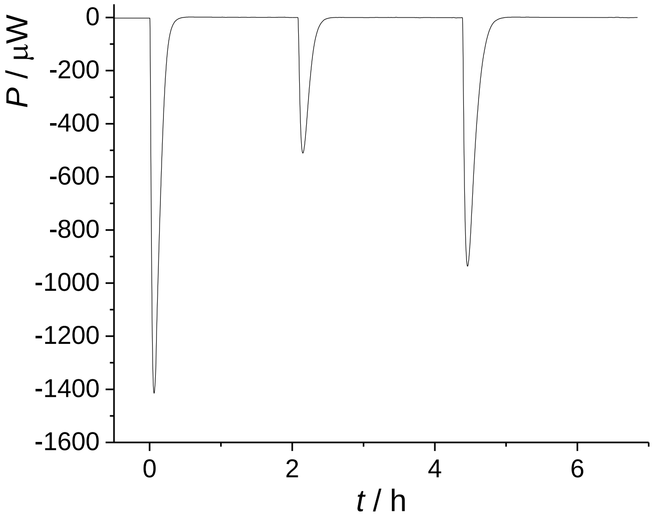

Figure S26. Calorimetric determination of dissolution enthalpy of **tpps** (m_1=7.08 mg, m_2=3.03 mg, m_3=6.35 mg) in acetonitrile at 25°C; stirring rate = 60 rpm.

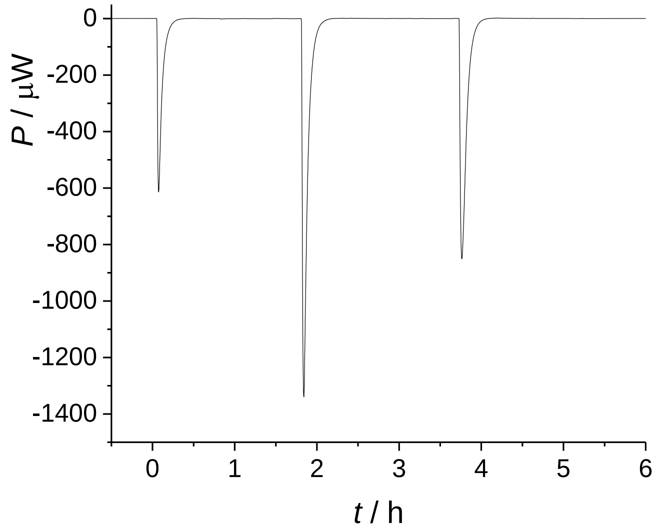

Figure S27. Calorimetric determination of dissolution enthalpy of (**tpps**)(**tftib**) (m_1=2.62 mg, m_2=6.12 mg, m_3=4.83 mg) in acetonitrile at 25°C; stirring rate = 60 rpm.

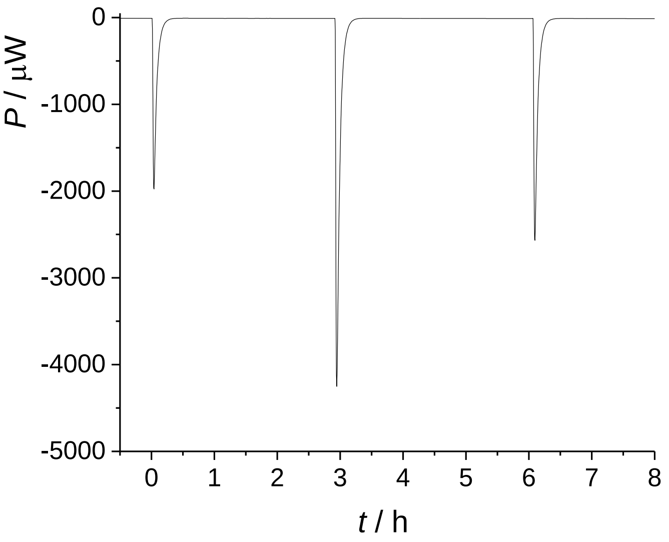

Figure S28. Calorimetric determination of dissolution enthalpy of **acr** (m_1=3.85 mg, m_2=8.42 mg, m_3=5.12 mg) in acetonitrile at 25°C; stirring rate = 60 rpm.

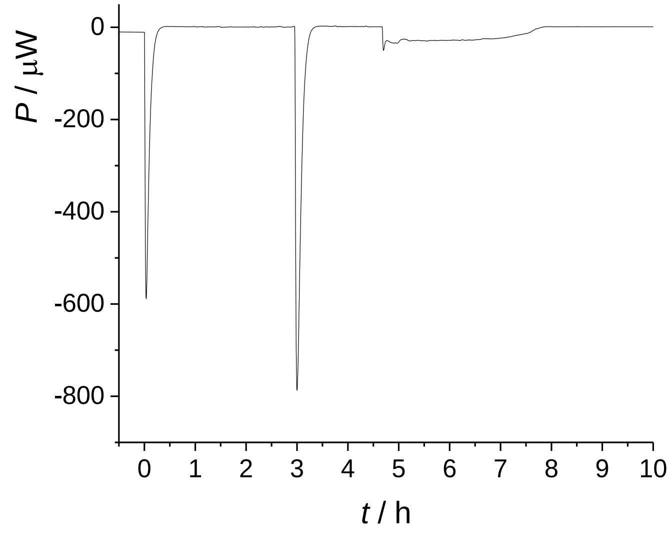

Figure S29. Calorimetric determination of dissolution enthalpy of (**acr**)(**tftib**) (m_1=3.02 mg, m_2=4.98 mg, m_3=4.28 mg) in acetonitrile at 25°C; stirring rate = 60 rpm.

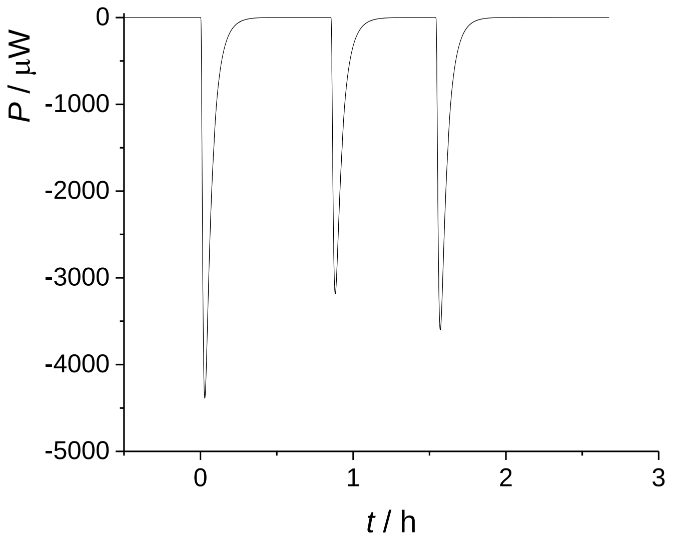

Figure S30. Calorimetric determination of dissolution enthalpy of **tmpyr** (m_1=6.26 mg, m_2=4.88 mg, m_3=5.22 mg) in acetonitrile at 25°C; stirring rate = 60 rpm.

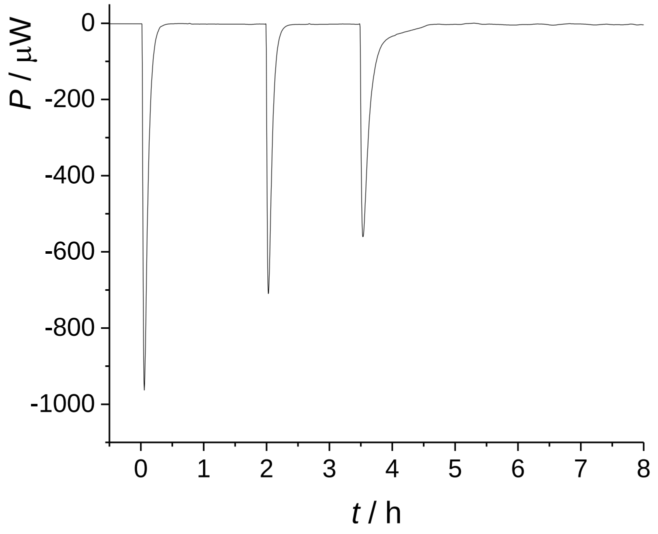

Figure S31. Calorimetric determination of dissolution enthalpy of (**tmpyr**)(**tftib**) (m_1=3.72 mg, m_2=2.87 mg, m_3=4.24 mg) in acetonitrile at 25°C; stirring rate = 60 rpm.

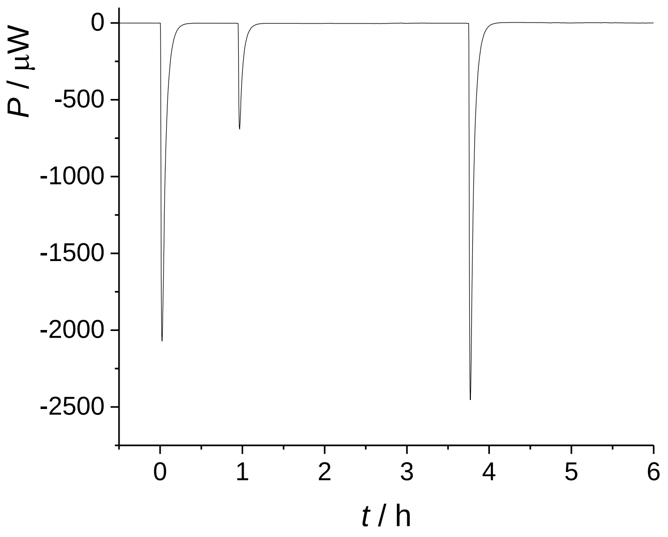

Figure S32. Calorimetric determination of dissolution enthalpy of **14tfib** (m_1=10.30 mg, m_2=3.77 mg, m_3=11.53 mg) in acetonitrile at 25°C; stirring rate = 60 rpm.

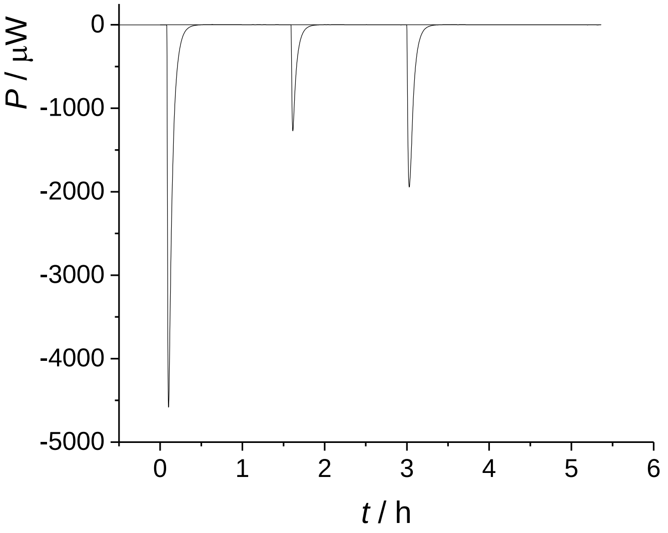

Figure S33. Calorimetric determination of dissolution enthalpy of (**pyr**)(**14tfib**) (m_1=10.35 mg, m_2=2.91 mg, m_3=5.21 mg) in acetonitrile at 25°C; stirring rate = 60 rpm.

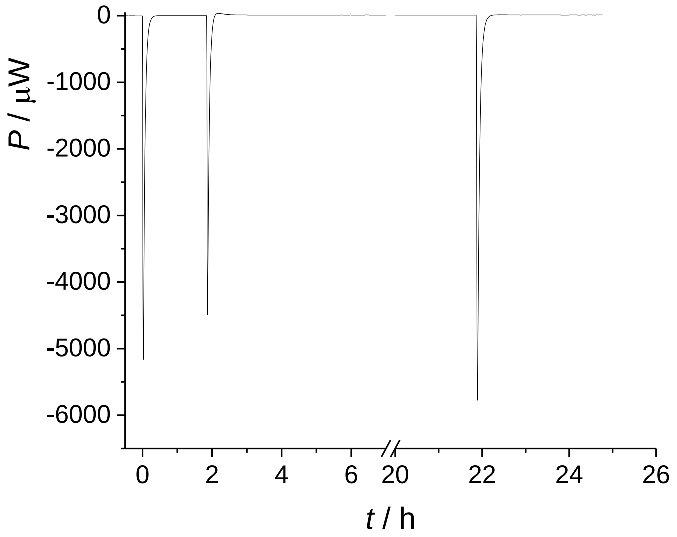

Figure S34. Calorimetric determination of dissolution enthalpy of **pyr** (m_1=6.08 mg, m_2=9.12 mg, m_3=6.82 mg) in acetonitrile at 25°C; stirring rate = 60 rpm.

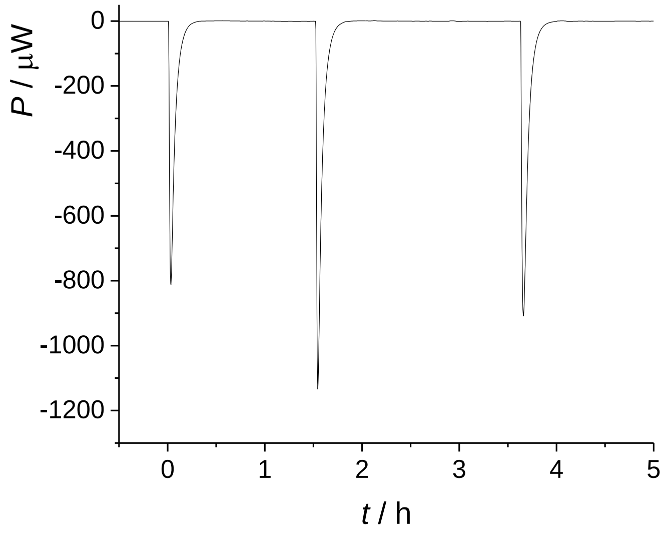

Figure S35. Calorimetric determination of dissolution enthalpy of (**pyr**)$_{1/2}$(**tftib**) (m_1=3.09 mg, m_2=4.44 mg, m_3=4.11 mg) in acetonitrile at 25°C; stirring rate = 60 rpm.

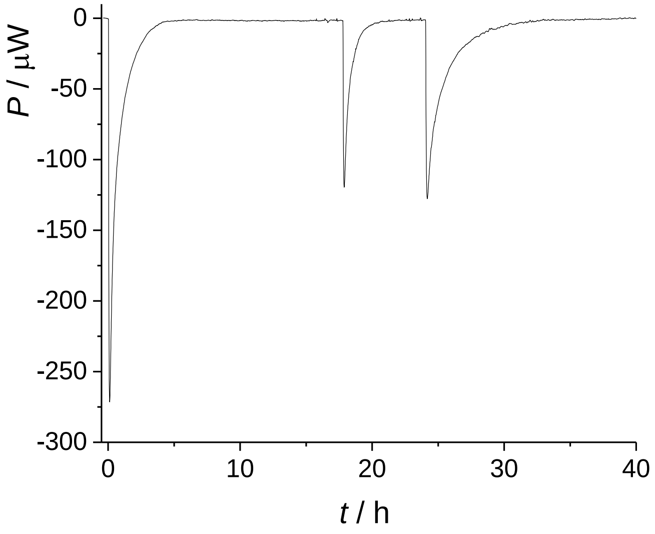

Figure S36. Calorimetric determination of dissolution enthalpy of **fum** (m_1=2.990 mg, m_2=0.965 mg, m_3=2.700 mg) in acetonitrile at 25°C; stirring rate = 60 rpm.

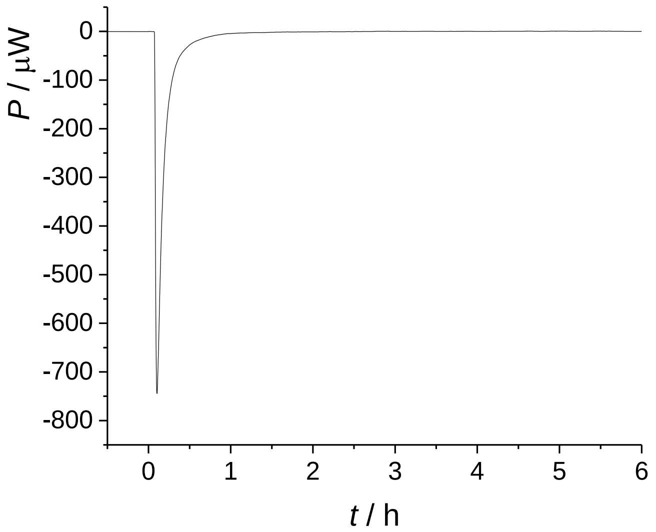

Figure S37. Calorimetric determination of dissolution enthalpy of (**pyr**)(**fum**) (m_1=1.200 mg) in acetonitrile at 25°C; stirring rate = 60 rpm.

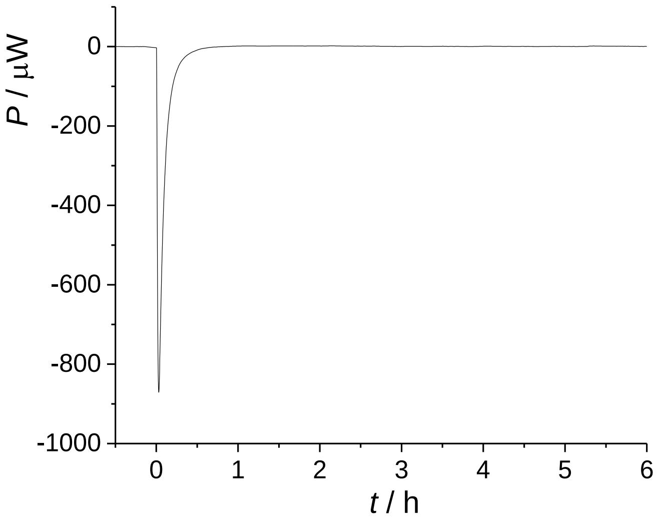

Figure S38. Calorimetric determination of dissolution enthalpy of (**pyr**)(**fum**) (m_1=1.145 mg) in acetonitrile at 25°C; stirring rate = 60 rpm.

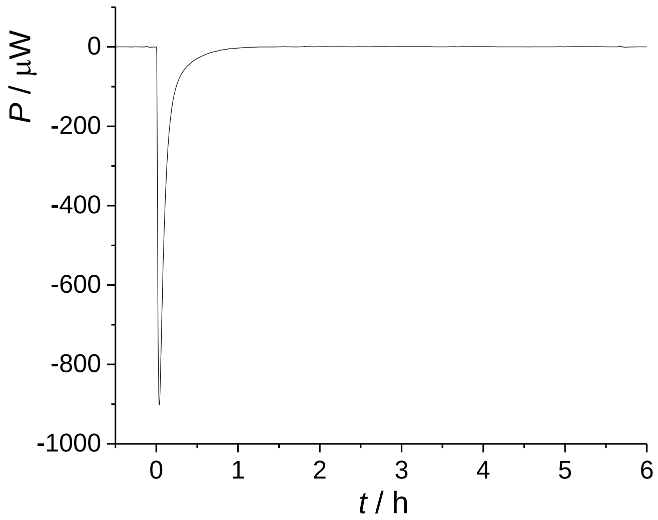

Figure S39. Calorimetric determination of dissolution enthalpy of (**pyr**)(**fum**) (m_1=1.430 mg) in acetonitrile at 25°C; stirring rate = 60 rpm.

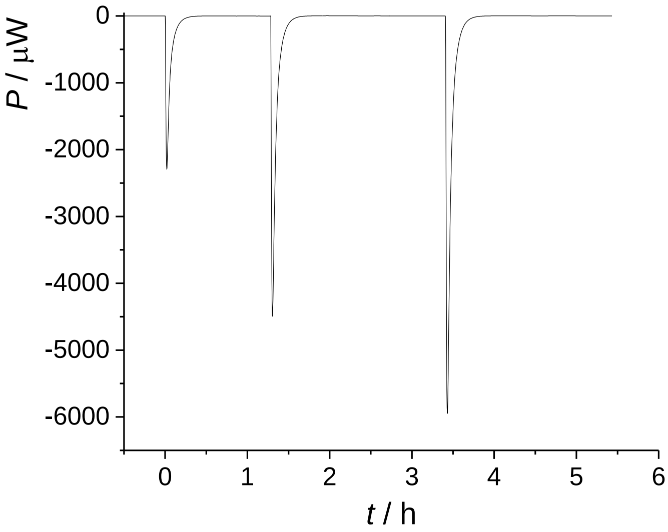

Figure S40. Calorimetric determination of dissolution enthalpy of **ox** (m_1=2.200 mg, m_2=5.340 mg, m_3=6.705 mg) in acetonitrile at 25°C; stirring rate = 60 rpm.

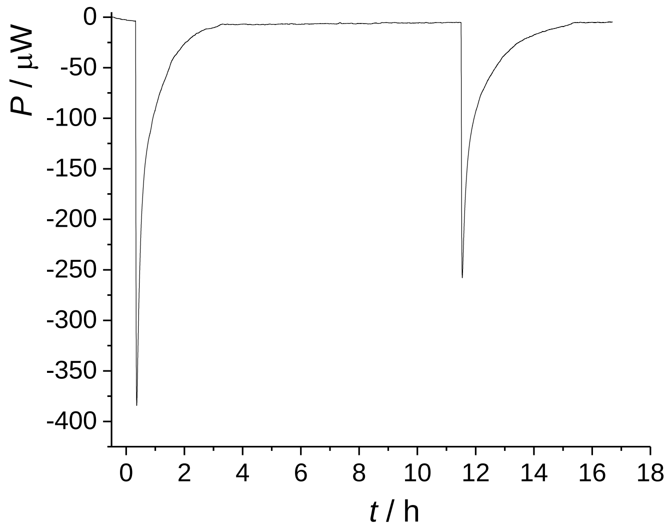

Figure S41. Calorimetric determination of dissolution enthalpy of (**pyr**)(**ox**) (m_1=1.910 mg, m_2=1.705 mg) in acetonitrile at 25°C; stirring rate = 60 rpm.

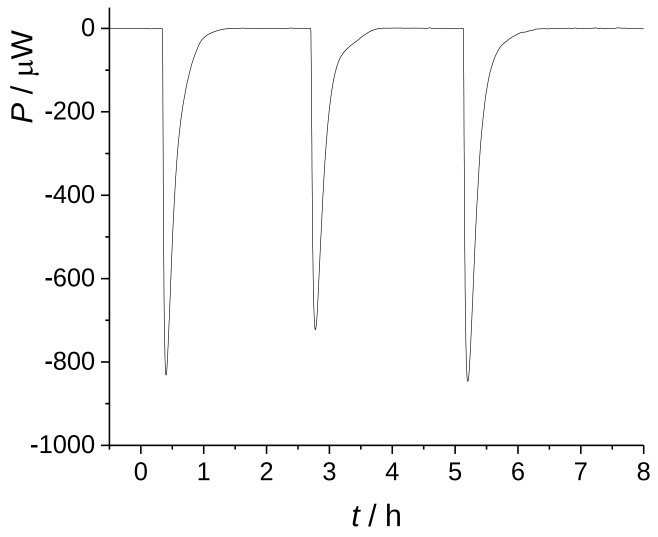

Figure S42. Calorimetric determination of dissolution enthalpy of **pht** (m_1=4.48 mg, m_2=4.09 mg, m_3=4.95 mg) in acetonitrile at 25°C; stirring rate = 60 rpm.

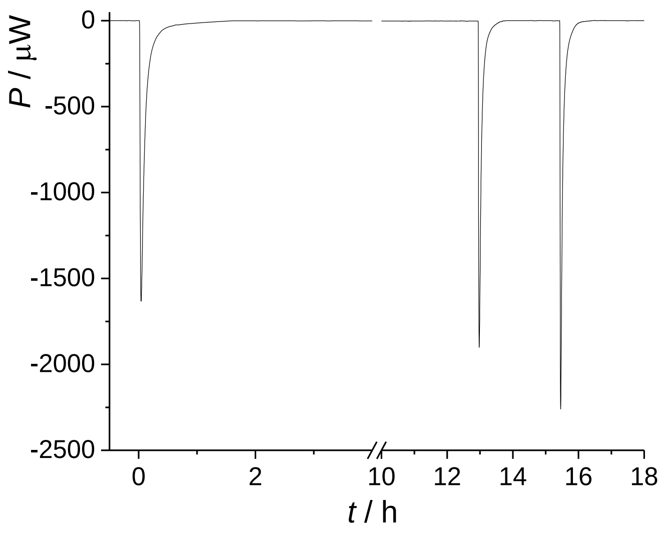

Figure S43. Calorimetric determination of dissolution enthalpy of (**pyr**)(**pht**) (m_1=3.51 mg, m_2=4.07mg, m_3=4.16 mg) in acetonitrile at 25°C; stirring rate = 60 rpm.

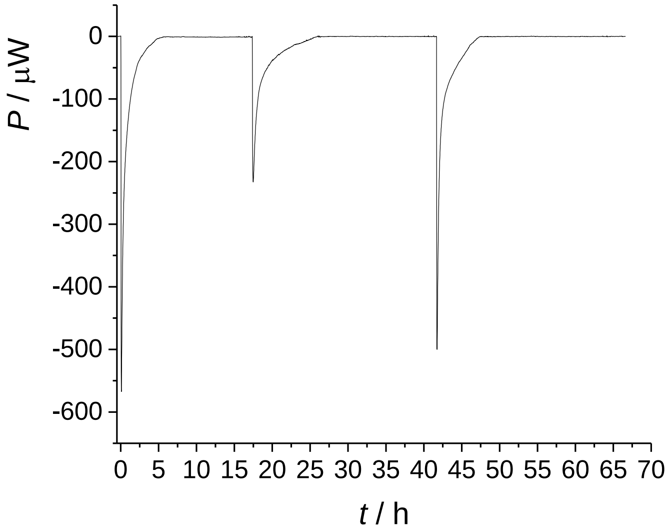

Figure S44. Calorimetric determination of dissolution enthalpy of **succ** (m_1=6.07 mg, m_2=4.86 mg, m_3=5.89 mg) in acetonitrile at 25°C; stirring rate = 60 rpm.

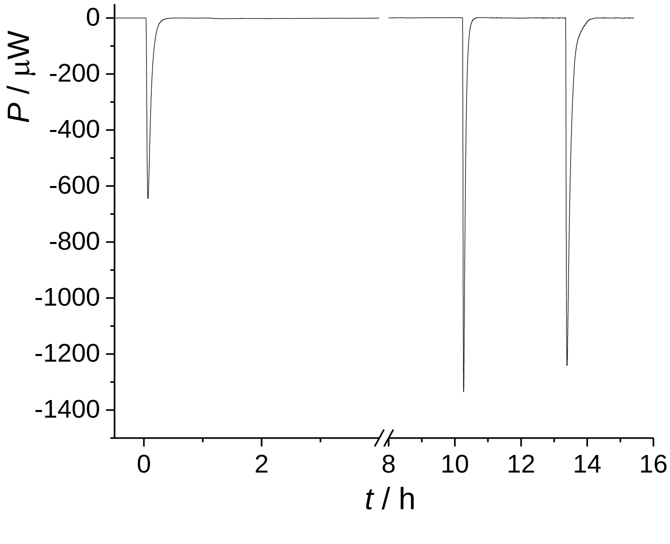

Figure S45. Calorimetric determination of dissolution enthalpy of (**pyr**)(**succ**) (m_1=0.645 mg, m_2=1.335, m_3=2.315 mg) in acetonitrile at 25°C; stirring rate = 60 rpm.

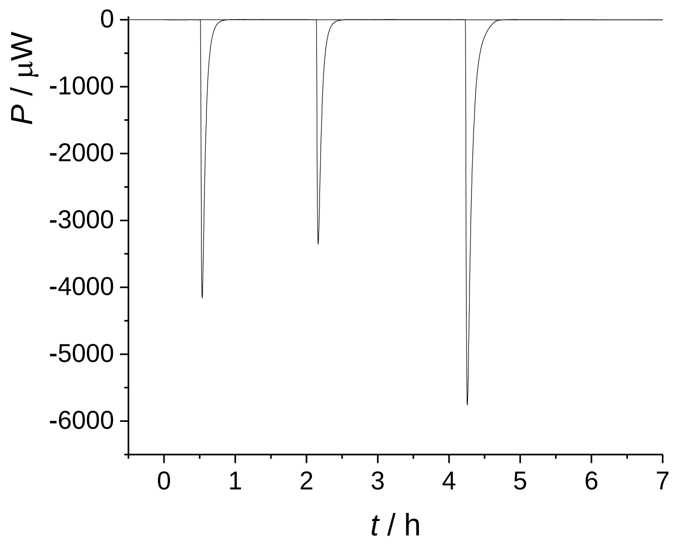

Figure S46. Calorimetric determination of dissolution enthalpy of **tdec** (m_1=3.70 mg, m_2=3.16 mg, m_3=6.78 mg) in acetonitrile at 25°C; stirring rate = 60 rpm.

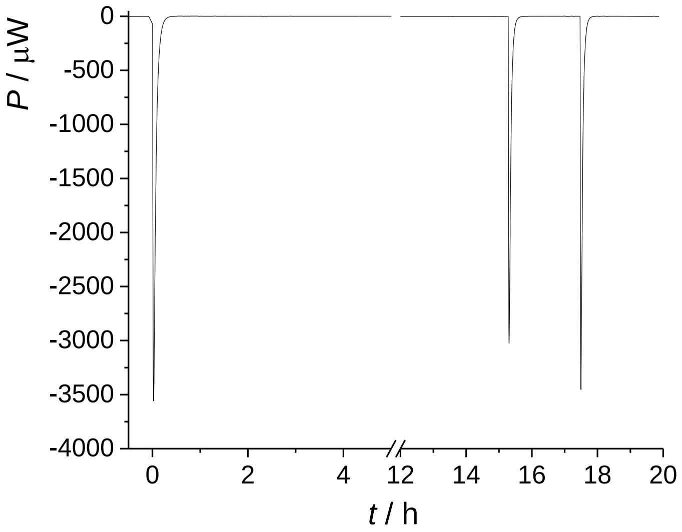

Figure S47. Calorimetric determination of dissolution enthalpy of (**pyr**)(**tdec**)$_2$ (m_1=3.14 mg, m_2=2.91 mg, m_3=3.10 mg) in acetonitrile at 25°C; stirring rate = 60 rpm.

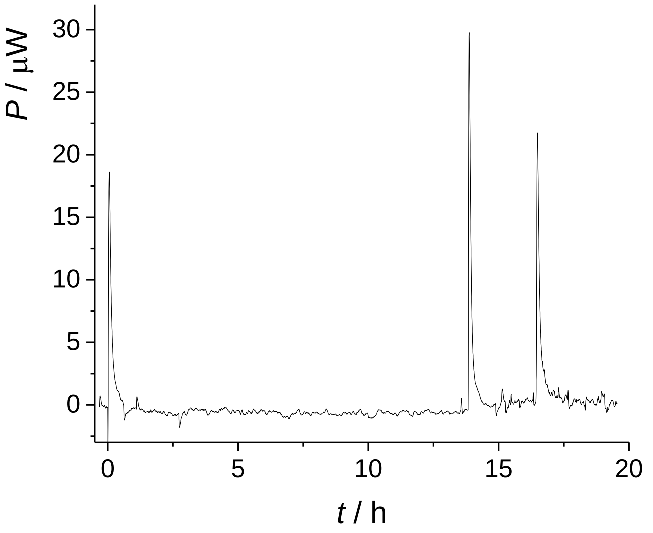

Figure S48. Blank experiments in acetonitrile at 25°C; stirring rate = 60 rpm.

Table S8. Dissolution enthalpies of studied compounds in acetonitrile at 25 °C. Uncertainties of the last digit are given in parentheses as standard errors of the mean.

compound	*m* / mg	*Q* / mJ	$\Delta_s H$ / kJ mol^{-1}
**tftib**	4.71	152.04	**17.4(1)**
	7.15	228.91	
	9.84	321.04	
**2,2’-bipy**	3.93	551.28	**21.9(4)**
	4.61	610.86	
	4.37	608.14	
**(2,2’-bipy)(tftib)**	1.640	123.41	**53.8(5)**
	1.035	68.399	
**4,4’-bipy**	2.58	308.16	**19.6(2)**
	5.10	623.51	
	5.80	728.44	
**(4,4’-bipy)(tftib)**	1.755	144.22	**59.0(3)**
	1.130	90.201	

Table S8. Continuation

**dabco**	5.23	883.12	**19.6(5)**
	3.01	527.04	
**(dabco)(tftib)**	3.79	327.61	**55.8(3)**
	5.02	440.00	
	4.02	346.98	
**(tpps)(tftib)**	2.62	138.92	**46.4(4)**
	6.12	344.91	
	4.83	268.97	
**tpps**	7.08	697.89	**29.0(2)**
	3.03	281.69	
	6.35	607.93	
**acr**	3.85	476.89	**22.70(1)**
	8.42	1053.4	
	5.12	636.17	
**(acr)(tftib)**	3.02	201.62	**48.6(1)**
	4.98	336.75	
	4.28	291.39	
**tmpyr**	6.26	1110.8	**24.1(3)**
	4.88	828.40	
	5.26	934.47	
**(tmpyr)(tftib)**	3.72	316.10	**57.5(4)**
	2.87	244.39	
	4.24	365.78	
**14tfib**	10.3	474.30	**18.1(8)**
	3.77	139.97	
	11.53	532.47	
**(pyr)(14tfib)**	10.35	1075.2	**51.1(7)**
	2.91	289.28	
	5.21	556.22	
**pyr**	6.08	1234.6	**16.49(5)**
	5.12	1043.7	
	6.82	1394.6	
**(pyr)$_{1/2}$(tftib)**	3.09	188.52	**34.5(5)**
	4.44	257.33	
	4.11	244.63	
**fum**	2.990	768.72	**30.4(1)**
	0.965	240.59	
	2.700	691.53	
**(pyr)(fum)**	1.200	334.70	**56.29(8)**
	1.145	317.33	
	1.440	403.11	

Table S8. Continuation

**ox**	2.200	494.87	**19.3(8)**
	5.340	1044.6	
	6.705	1421.7	
**(pyr)(ox)**	1.910	289.17	**57.0(3)**
	1.705	375.86	
**(pyr)(pht)**	3.51	646.32	**46(1)**
	4.07	717.54	
	4.16	795.84	
**pht**	4.48	671.84	**25.5(1)**
	4.09	619.44	
	4.95	744.48	
**succ**	6.07	1470.1	**28.82(8)**
	4.86	1166.6	
	5.89	1432.4	
**(pyr)(succ)**	0.645	192.04	**60.5(1)**
	1.335	391.55	
	2.315	677.00	
**tdec**	3.70	1059.8	**61.7(5)**
	3.16	880.31	
	6.78	1963.3	
**(pyr)(tdec)$_2$**	3.14	899.20	**148.3(5)**
	2.91	834.29	
	3.16	913.48	

S5 References

1 S. J. Clark, M. D. Segall, C. J. Pickard, P. J. Hasnip, M. I. J. Probert, K. Refson and M. C. Payne, *Z. Kristallogr.*, 2005, **220**, 567–570.

2 C. R. Groom, I. J. Bruno, M. P. Lightfoot and S. C. Ward, *Acta Crystallogr. B.*, 2016, **B72**, 171–179.

3 T. Björkman, *Comput. Phys. Commun.*, 2011, **182**, 1183–1186.

4 J. P. Perdew, K. Burke and M. Ernzerhof, *Phys. Rev. Lett.*, 1996, **77**, 3865–3868.

5 S. Grimme, J. Antony, S. Ehrlich and H. Krieg, *J. Chem. Phys.*, 2010, **132**, 154104.

6 A. Tkatchenko, R. A. DiStasio, R. Car and M. Scheffler, *Phys. Rev. Lett.*, 2012, **108**, 236402.

7 A. Ambrosetti, A. M. Reilly, R. A. DiStasio and A. Tkatchenko, *J. Chem. Phys.*, 2014, **140**, 18A508.

8 A. M. Reilly and A. Tkatchenko, *Chem. Sci.*, 2015, **6**, 3289–3301.

9 H. J. Monkhorst and J. D. Pack, *Phys. Rev. B*, 1976, **13**, 5188–5192.

10 H. M. Rietveld, *J. Appl. Crystallogr.*, 1969, **2**, 65–71.

11 A. A. Coelho, *J. Appl. Crystallogr.*, 2018, **51**, 210–218.

12 W. A. Dollase, *J. Appl. Crystallogr.*, 1986, **19**, 267–272.

13 CrysAlis CCD and CrysAlis RED, Oxford Diffraction, Oxford Diffraction Ltd: Yarnton, 2008.

14 G. M. Sheldrick, *Acta Crystallogr. A.*, 2008, **A64**, 112–22.

15 O. V. Dolomanov, L. J. Bourhis, R. J. Gildea, J. A. K. Howard and H. Puschmann, *J. Appl. Crystallogr.*, 2009, **42**, 339–341.

16 C. F. Macrae, I. J. Bruno, J. A. Chisholm, P. R. Edgington, P. McCabe, E. Pidcock, L. Rodriguez-Monge, R. Taylor, J. van de Streek and P. A. Wood, *J. Appl. Crystallogr.*, 2008, **41**, 466–470.
